# Liposomes-Based Drug Delivery Systems of Anti-Biofilm Agents to Combat Bacterial Biofilm Formation

**DOI:** 10.3390/antibiotics12050875

**Published:** 2023-05-08

**Authors:** Zinb Makhlouf, Amaal Abdulraqeb Ali, Mohammad Hussein Al-Sayah

**Affiliations:** 1 Department of Biology, Chemistry and Environmental Sciences, American University of Sharjah, Sharjah P.O. Box 26666, United Arab Emirates; 2 Biomedical Engineering Program, American University of Sharjah, Sharjah P.O. Box 26666, United Arab Emirates

**Keywords:** liposomes, drug delivery, antibiotics, biofilms, antimicrobial, *Pseudomonas aeruginosa*, *Escherichia coli*, *Staphylococcus aureus*

## Abstract

All currently approved antibiotics are being met by some degree of resistance by the bacteria they target. Biofilm formation is one of the crucial enablers of bacterial resistance, making it an important bacterial process to target for overcoming antibiotic resistance. Accordingly, several drug delivery systems that target biofilm formation have been developed. One of these systems is based on lipid-based nanocarriers (liposomes), which have shown strong efficacy against biofilms of bacterial pathogens. Liposomes come in various types, namely conventional (charged or neutral), stimuli-responsive, deformable, targeted, and stealth. This paper reviews studies employing liposomal formulations against biofilms of medically salient gram-negative and gram-positive bacterial species reported recently. When it comes to gram-negative species, liposomal formulations of various types were reported to be efficacious against *Pseudomonas aeruginosa*, *Escherichia coli*, *Acinetobacter baumannii*, and members of the genera *Klebsiella*, *Salmonella*, *Aeromonas*, *Serratia*, *Porphyromonas*, and *Prevotella*. A range of liposomal formulations were also effective against gram-positive biofilms, including mostly biofilms of *Staphylococcal* strains, namely *Staphylococcus aureus*, *Staphylococcus epidermidis*, and *Staphylococcus saprophyticus* subspecies *bovis*, followed by *Streptococcal* strains (*pneumonia*, *oralis*, and *mutans*), *Cutibacterium acnes*, *Bacillus subtilis*, *Mycobacterium avium*, *Mycobacterium avium *subsp.* hominissuis*, *Mycobacterium abscessus*, and *Listeria monocytogenes* biofilms. This review outlines the benefits and limitations of using liposomal formulations as means to combat different multidrug-resistant bacteria, urging the investigation of the effects of bacterial gram-stain on liposomal efficiency and the inclusion of pathogenic bacterial strains previously unstudied.

## 1. Introduction

From walls, floors, drains, plastic wares, glass surfaces, food, oral cavities, intestines, contact lenses, vascular grafts, to heart valves, bacteria have established their biofilms in an extensive list of biotic and abiotic environments [1,2]. This surface attachment in an extracellular polymeric substance matrix enables bacteria to exchange DNA and thrive despite famine, desiccation, mechanical forces, unfavorable pH, and even ultraviolet radiation [1,2]. Moreover, unlike their planktonic counterpart, bacteria in biofilms are up to 1000-fold more resistant to antibiotic agents [2,3]. Hence, when it comes to the impending arrival of the “post-antibiotic era”, bacterial biofilm formation is one of the foundational facilitators [4]. In fact, approximately 65–80% of all microbial infections and 80% of human chronic infections are brought about by bacterial biofilm formation [5,6]. Despite the preceding, the development of new antibiotics is far from keeping up with the emerging bacterial resistance, where currently, an approved anti-biofilm agent does not exist. Eventually, it is anticipated that infections brought about by antibiotic-resistant bacteria will become the number one cause of death by 2050 [4,5,7,8]. Furthermore, all currently approved natural, semi-synthetic, or synthetic antibiotic classes have been met with resistance by at least some of the pathogens they target [9]. Accordingly, there is a pressing need for novel therapeutic measures that effectively target bacterial biofilms and impede the development of bacterial resistance.

Since biofilm penetration is a markedly challenging hurdle to overcome, nanotechnology-derived antimicrobial delivery-systems are gaining more appreciation [8]. While there are various ways to classify nanoparticles, such as shape or size, they are generally classified as organic (dendrimers, micelles, liposomes, and ferritin), inorganic (metal and metal oxide based), carbon-based (fullerenes, graphene, carbon nano tubes, carbon nanofibers, and carbon black), and ceramic nanoparticles [10,11,12]. Analogs of cell membranes, liposomes are lipid-based organic nanocarriers whose hydrophilic lipid heads and hydrophobic tails determine their surface properties and fluidity, respectively [8,10]. These nanocarriers can encapsulate a wide variety of drugs due to their amphiphilic nature, as their aqueous compartment bears hydrophilic drugs while their lipid bilayer accommodates hydrophobic drugs [13]. Notably, various studies report the efficacy, stability, and safety of liposome-encapsulated antibiotics [13,14,15]. Herein, this review discusses advances in liposomal-antibiofilm agent delivery and the efficacy of liposome-encapsulated antibiofilm agents against pathogenic gram-negative and gram-positive bacteria.

## 2. Bacterial Biofilms

Bacterial biofilms are aggregates of one or more bacterial species attached to a surface, protected by a self-produced matrix consisting of polysaccharides, proteins, glycoproteins, and nucleic acids [16,17]. Common species that form biofilms include *Pseudomonas aeruginosa* (*P. aeruginosa*), *Staphylococcus epidermidis* (*S. epidermidis*), *Enterococcus faecalis* (*E. faecalis*), *Staphylococcus aureus* (*S. aureus*), *Klebsiella pneumoniae* (*K. pneumoniae*), *Streptococcus viridans* (*S. viridans*), *Escherichia coli* (*E. coli*), and *Proteus mirabilis* (*P. mirabilis*) [18]. Biofilm-encapsulated bacteria grow very slowly and in close contact to exchange genetic material and communicate chemically, which regulates their gene expression profile with respect to the microbial population’s size, a process also known as quorum sensing [19]. Encapsulation within the extracellular polymeric matrix allows bacteria to thrive in harsh conditions, such as the administration of sanitizers and antibiotics [19]. Aside from the physical protection provided by this matrix, bacteria in biofilms evade the immune system and anti-microbial agents by staying dormant and employing various adaptations to withstand nutrient scarcity and environmental anoxia [20]. Moreover, these bacteria alter their metabolism, gene expression, and protein production, hence, reducing cellular functions that can be targeted by antibiotics [20]. Additionally, due to the skewed balance between the external oxygen supply and the internal oxygen consumption, biofilms are characterized by hypoxia which contributes to the antibiotic resistance of sessile bacteria [16]. Biofilms also encompass phenotypic variants with marked antibiotic resistance that, unlike resistant cells, do not grow in the presence of antibiotics but instead remerge when their levels drop, known as “persister cells” [21].

While biofilms are observed extracellularly, bacteria may also establish their biofilms inside living cells [20]. Regardless, the transformation of planktonic bacterial cells to their sessile form follows the same steps. A reversible attachment to a conditioned surface (optimally rough, hydrophobic, and coated with different organic substances) is mediated by very weak interactions, namely van der Waals and hydrophobic interactions [19]. Next, this reversible attachment strengthens and becomes irreversible [22,23]. The stabilization of this attachment is then ensued by microbial cell proliferation and the formation of micro-colonies, which then proceed to produce the extra polymeric matrix through quorum sensing, mediating its formation and maturation [22,23]. The resulting mature matrix includes small channels that carry nutrients, water, and waste [4]. Finally, bacteria spread from the matured biofilm and commence new cycles [23].

Therapeutic options for biofilm infections are scarce, as biofilms are not treated or eradicated with ease [24]. Currently, antibiofilm agents employ one of these strategies: biofilm formation inhibition, biofilm dispersal, or biofilm eradication. Biofilm inhibition is mediated by preventing bacterial attachment through modifying surface properties such as hydrophobicity, texture, and roughness using antibiotics, metal ions, or other synthetic compounds [25]. Biofilm dispersal is mainly mediated by targeting key chemical pathways that are biofilm-maintaining, such as quorum sensing, which enhances the potency of prescribed antimicrobial agents [24,26]. Biofilm eradication is carried out by eradicating cells residing in biofilms and is achieved by antibiotic administration, namely antimicrobial peptides and lipids, quaternary ammonium compounds, and nitric oxide-releasing antibiotics [26]. Despite the preceding, there is a lack of clinically approved anti-biofilm agents [5,27]. This is plausible given that no standardized evaluation method of biofilm inhibition and/or eradication capacity of novel compounds exists [27]. Moreover, while advances have been made in the quest for antibiofilm agents, clinical studies remain lacking [28]. Hence, the quest for novel antibiofilm agents is of paramount importance, especially since the current agents with observed antibiofilm activity also display cytotoxic effects [29].

## 3. Challenges in Targeting Biofilms

Various factors make targeting biofilms a difficult objective. Firstly, the failure of antimicrobial agents to directly and specifically target biofilms demands the administration of high dosages to achieve desired effects. This is plausible, as the biofilm extracellular polymeric matrix shields and limits the agent’s access to biofilm-bound bacteria, demanding drug dosages up to 1000-fold higher than that of targeting planktonic bacterial cells [15,30]. Accordingly, the possibility of tissue damage as a result of drug toxicity is heightened. Therefore, localized delivery of antimicrobials to biofilms is needed. Furthermore, the nullification of the antibiotic activity of anti-biofilm agents by the immune system presents another hurdle to overcome. Thus, a method to veil the anti-biofilm agent from the immune system is needed [15]. However, even if the anti-biofilm agent does succeed in penetrating the biofilm, the acquired resistance of bacterial cells to previously used antibiotics undermines the agent’s success. In fact, this has rendered many of the currently employed agents ineffective [15,30,31]. Further slimming the chances of success, the heterogeneity among biofilm layers arising from heterogeneity in growth and metabolic activity poses another challenge. Moreover, this heterogeneity resulting from the different conditions, such as oxygen levels and pH within biofilm layers, may affect the activity of the agent [15]. Accordingly, the characteristics of promising anti-biofilm agents are biocompatibility, selective targeting, non-immunogenicity, stability, and biofilm penetration. Notably, nano-sized drug delivery is one of the systems that have gained appraisal for the delivery of antibacterial agents due to their biocompatibility, stability, tunable size, and easy surface functionalization, which makes them excellent candidates for anti-biofilm agents [15,32].

## 4. Liposomes: Versatile Drug Delivery Systems

**Figure 1:**
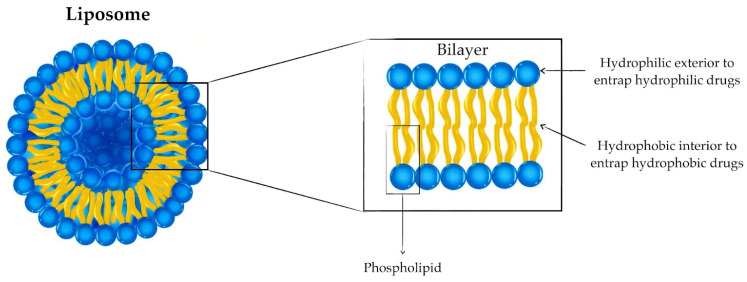
Structure of liposomes to entrap hydrophilic and hydrophobic drugs.

Antibiotics can be delivered to biofilms via conjugation to, or encapsulation within, the nano-drug delivery system. Encapsulation is particularly beneficial as it shelters the enclosed agent from potential inactivation and degradation, and reduces its associated toxicity and side effects. Liposomes are one of the most promising and commonly studied nano-vehicles to carry and deliver antimicrobial agents. This is mainly due to their ability to carry a wide range of antibiotics and infiltrate the extra polymeric matrix [15]. Liposomes are spherical vesicles of diameters typically between 50 and 500 nm with a hydrophilic interior formed by the self-assembly of lipids into a lipid bilayer. Liposomes can carry hydrophilic and hydrophobic cargo within their inner cavity and the bilayer itself, respectively, as shown in Figure 1 [8,15,33].

Liposomes can be formulated from a wide range of amphiphilic lipids, including phospholipids and glycolipids, which determine the properties of the liposome. While the surface properties of liposomes are determined by the lipid’s polar heads, the fluidity of the membrane is dictated by the nonpolar portion of the lipid. A major advantage of liposomes for delivery purposes is their similarity to biological cell membranes. In antimicrobial applications, this advantage facilitates the fusion of the liposome to the bacterial membrane, allowing the delivery of the antibiotic into the cytoplasm of the bacteria [8,15,34].

Due to their advantageous properties, different types of liposomes have been explored to perforate bacterial biofilms, improve antibiotic delivery, reverse antibiotic resistance, and inhibit growth in an array of gram-positive and -negative strains [35,36,37,38,39,40]. The liposomal types investigated for biofilm treatment can be classified according to composition into conventional first-generation liposomes (neutral, cationic, or anionic), stealth (PEGylated) liposomes, actively targeted liposomes, stimuli-responsive liposomes, and bubble liposomes [33,41]. In addition, liposomes can also be classified based on their size and number of bilayers into unilamellar vesicles (small and large), multilamellar vesicles, and oligolamellar vesicles [42]—the different types of liposomes and their advantages and disadvantages as delivery systems are discussed below.

Conventional liposomes are non-modified liposomes in which the lipid bilayer comprises cationic, anionic, or neutral phospholipids together with cholesterol (Figure 1). Although proving advantageous in reducing the toxicity of the liposome-encapsulated drug, conventional liposomes as therapeutic drug carriers are limited by their facile clearance from the bloodstream by macrophages of the reticuloendothelial system post-opsonization [41,42]. This elimination is more pronounced for cationic and larger liposomes due to the electrostatic interaction between the positively charged liposomes and the negatively charged biological macromolecules [42,43]. In terms of antibacterial and anti-biofilm activities, cationic liposomes tend to be more effective compared to negative or neutral liposomes of the same size [34]. The cationic antimicrobial particles may reduce bacterial adhesions, thereby disrupting the formation of the biofilm [44]. This is possibly due to the electrostatic attractive forces between the positively charged liposomes and the negative surface of the bacteria/biofilm [15,45]. Furthermore, first-generation liposomes lack any active targeting mechanisms and utilize only passive targeting to localize the vehicle within tissues having a discontinuous endothelial lining [42].

Despite their advantages, the efficiency of conventional liposomes is limited for applications involving skin penetration due to their rigidity. Therefore, deformable (or elastic) liposomes have been developed to enhance drug delivery across the skin membrane. Due to their higher elasticity that surpasses that of conventional liposomes, deformable liposomes are more suitable for skin penetration into deeper layers of the epidermis [46,47].

Furthermore, to prolong their circulation time, sterically stabilized liposomes with longer blood circulation time and protection from macrophages were developed. One of the most commonly studied stabilized liposomes are polyethylene glycol (PEG) liposomes, also called stealth liposomes. PEG is a non-ionic hydrophilic polymer commonly used on the surfaces of drug delivery systems to mask them from opsonization and hence, elimination by the reticuloendothelial system. Furthermore, PEG coating prevents aggregation of the liposomes via steric stabilization [42,48]. PEGylated liposomes have been extensively reported as superior alternatives to conventional liposomes in terms of blood circulation [49,50,51,52,53]. However, stealth liposomes suffer from disadvantages, including their potential hypersensitivity (which depends on several parameters such as size) and their non-biodegradability, which restricts their use to low molecular weight PEGs [48].

To solve the issue of the non-specificity of conventional liposomes, actively targeted liposomes have been established in which the liposome surface is modified with a moiety, such as antibodies that can target a specific tissue or organ in the body. For instance, immunoliposomes (antibody-modified liposomes) have been commonly reported for efficient antibody-mediated targeting for applications including biofilm treatment [54,55,56,57,58,59]. However, bare immunoliposomes without steric stabilization are inefficient due to their rapid elimination. Hence, immunoliposomes are usually modified with PEG to achieve targeting while avoiding rapid clearance from circulation [54]. Other ligands used to target liposomes include aptamers [60,61] and peptides [62].

Stimuli-responsive liposomes are another type of liposome attracting extensive attention due to their ability to respond to certain stimuli and achieve a controlled and targeted drug release. Exploiting stimuli for targeted drug release is especially beneficial as it reduces the risk of off-target drug toxicity [63,64]. Such stimuli responsiveness can be achieved by modifying the lipids making up the bilayer to respond to endogenous or external stimuli [43]. Endogenous (or internal) stimuli include internal conditions and triggers such as redox conditions or pH, while exogenous (or external) stimuli include externally applied triggers such as light and ultrasound [63]. Although endogenous stimuli have been reported with other nanoparticles, none have been used with liposomes. As for exogenous stimuli, the biofilm microenvironment differs from normal tissue environments in its low pH, elevated H_2_O_2_ levels, overexpression of some enzymes, and accumulation of the thiol glutathione (GSH). The presence of such distinct environmental features within the biofilm makes it possible to use those internal conditions as triggers for the biofilm-specific release of anti-biofilm agents [65]. To benefit from exogenous stimuli, liposomes have been combined with other nanoparticles that can respond to externally applied triggers. For example, thermo-sensitive liposomes have been combined with gold nanoparticles which respond to near-infrared light by generating heat. The generated heat causes the liposome to undergo a phase transition and become more fluidic, thereby releasing encapsulated cargo [66]. Exogenous stimuli, such as near-infrared irradiation, have been reported to trigger drug release from liposomes for biofilm treatment, as discussed in the upcoming section of this review [67,68,69].

Bubble liposomes utilize ultrasound to burst a gas bubble encapsulated within the liposomes together with the drug(s) to be delivered. Bursting of the bubble post-ultrasound application disrupts the liposome and leads to the controlled release of hosted drug(s) [70]. However, bubble liposomes are challenged by their low gas loading capacity and large sizes (from 500 nm to microns), and their costly and complicated preparation procedures [70]. Bubble liposomes have been explored for biofilm treatment, as reported by Fu, et al. [71] and Zhou, et al. [72].

Despite the advantages provided by each liposomal formulation, they still possess their own limitations. For instance, while conventional liposomes reduce drug-associated toxicities, they still suffer from their rapid removal from circulation [41]. This issue is solved by the introduction of PEG on the surface of liposomes. However, PEG coating is limited by the non-biodegradability of high-molar-mass PEG and the toxicity of low-molar-mass PEG. Therefore, it is important to ensure that the molar mass of PEG does not surpass the limit of renal elimination. Further adding to the issue is the difficulty in determining PEG’s threshold of renal elimination [48]. Due to their rigidity, conventional liposomes also face a challenge in penetrating the skin barrier in the case of topical applications. This issue is overcome using elastic (deformable) liposomes, which have better skin penetration due to their higher elasticity [47]. Nevertheless, deformable liposomes also suffer from limitations due to their prolonged elasticity and deformability; thus, these liposomes are typically unstable over extended storage periods. This, in turn, results in the loss of the liposome content during the storage period, which in turn impedes upscaling of the formulation [47]. Like conventional liposomes, targeted liposomes also suffer from rapid elimination from the bloodstream unless sterically stabilized (e.g., by PEG) [54].

On the other hand, non-specific release by liposomes can limit their therapeutic efficacy by reducing their bioavailability at the target site. To overcome such non-specificity, stimuli-responsive liposomes, which can release their cargo in response to internal and/or external stimuli, can be used [43,63,64]. However, the limitations of stimuli-responsive liposomes vary depending on the type of stimulus used. For instance, ultraviolet-responsive liposomes are limited due to ultraviolet’s poor tissue penetration and damage to cells/tissues [73]. Furthermore, it is important for the liposomes to have a high drug encapsulation efficiency, which can improve the bioavailability of the drug. However, encapsulation efficiency is dependent on the type of phospholipids making up the liposomes [74]. Therefore, it is important to ensure liposomal formulations for drug delivery, including antibiofilm agent delivery, are stable, have high encapsulation efficiency, are not rapidly eliminated from the bloodstream, and have high bioavailability (e.g., by targeting and stimuli-responsivity). The different types of liposomes are summarized in Table 1.

While other nano-drug delivery vehicles, such as metallic and polymeric nanoparticles, have been developed and studied, liposomes are particularly advantageous. This is due to their ability to carry and deliver hydrophilic and/or hydrophobic cargo, high biocompatibility, biodegradability, lack of immunogenicity and toxicity, and easy modification with targeting moieties. Furthermore, several liposome-based compositions have been approved by the FDA for the clinical treatment of infectious diseases. This indicates the promise of future liposomal formulations [13]. However, as mentioned in the previous paragraph and Table 1, liposomes do possess some limitations that need to be considered when designing a liposomal formulation for drug delivery purposes, including anti-biofilm agent delivery.

Throughout the next sections of this paper, the different liposome types are discussed for their effectiveness and potential in treating biofilms of clinically relevant gram-positive and gram-negative bacterial strains.

## 5. Liposomal Formulations for the Treatment of Gram-Negative Biofilms

Prominent agents behind global mortality and morbidity, gram-negative bacteria are less susceptible to antibiotic agents than gram-positive bacteria [75]. Unlike their gram-positive counterparts, gram-negative bacteria, except for the lipopolysaccharide (LPS)-deficient strains, are enveloped with LPS [76]. LPS serves as a stimulator of the immune system and a facilitator of antibiotic resistance due to its insulation of the bacterial cell it encapsulates [77]. In fact, LPS-deficient strains are less virulent and more susceptible to antibiotics [76]. Accordingly, it is no surprise that gram-negative bacteria constitute the majority of the WHO antibiotic-resistant pathogens list [75,77]. Some of the prominent gram-negative strains include *P. aeruginosa*, *E. coli*, *Acinetobacter baumannii* (*A. baumannii*), *Salmonella*, *Klebsiella*, *Serratia*, *Aeromonas*, and *Porphyromonas* spp. The following sections summarize recent studies implementing liposomal formulations against medically salient gram-negative bacteria. Table 2 summarizes all the studies investigating liposomal delivery systems for gram-negative anti-biofilm activity.

### 5.1. Pseudomonas aeruginosa Biofilms

*P. aeruginosa* is a multidrug-resistant gram-negative bacterium responsible for various human infections, ranging from urinary tract infections to septicemia [78,79]. While rarely affecting the immunocompetent, this bacterium acquired resistance to many antibiotics, namely aminoglycosides, quinolones, and β-lactams [80]. Currently, therapeutic options for *P. aeruginosa* infections are limited to combinational treatment and new antibiotics, some of which include mild to moderate side effects, and some still have not been objects of clinical trials such as POL7002 [80]. Regardless, therapeutic options are hindered by the bacterium’s notable ability to form compact biofilms, as none exist for biofilm infections [81,82]. Accordingly, there is a pressing need for novel anti-*P. aeruginosa* compounds that can target the bacterium’s biofilm. The efficacy of a variety of liposomal formulations against *P. aeruginosa* biofilms has been reported in the literature. Conventional charged and neutral liposomes have been observed to possess marked antibiofilm efficacy against *P. aeruginosa* [36,78,81,83,84]. In their study, Ibaraki and coworkers [81] investigate the anti-biofilm potential of surface modification by testing charged liposomes without or with polyethylene glycol (PEG) modification. Notably, they report marked antibiofilm activity and biofilm retention by cationic liposomes, and enhanced biofilm permeability by anionic liposomes. Nonetheless, these effects were amplified upon PEGylation. This could be elucidated by the enhanced permeability upon PEGylation and the amplified fusion of cationic liposomes with the naturally negatively charged biofilms. Other studies employing cationic liposomes bearing antibiotics confirm these findings [36,84]. Liposomal encapsulation has been reported to enhance the activity of various antimicrobials, yet that efficacy maybe lost due to the spontaneous fusion of liposomes [84]. Accordingly, Hou, et al., implemented a straightforward method for liposomal stabilization: lysozyme association, hence reducing its spontaneous fusion. Notably, the lysozyme-associated cationic liposomal formulation encapsulating gentamicin led to a marked reduction in biofilm mass in comparison to free gentamicin and lysozyme. Tobramycin is a frontline treatment for *P. aeruginosa* infections due to its inhibition of protein synthesis, yet even this antibiotic has been losing its efficacy against the bacterium [36]. Moreover, a below MIC of tobramycin has been reported to promote the formation of *P. aeruginosa* biofilm formation and drug-resistance [85]. Accordingly, the group envisioned encapsulating this antibiotic within cationic liposomes in conjugation with the cationic, synthetic, antibiofilm peptide: the innate defense regulator peptide-1018 (IDR-1018) [36]. Remarkably, encapsulation of the antibiotic within cationic liposomes engendered significant antibiofilm effects with or without IDR-1018. Of note, another work contradicts the previous findings, reporting superior antibiofilm effects of negatively charged liposomes in comparison with their neutral counterpart [78]. The authors explain that this discrepancy is brought about by the fact that the attraction of the positively charged agent to the negatively charged biofilm prevents its further penetration into the biofilm-bound bacteria, unlike its effects on sessile bacteria. Hence, the enhanced antibiofilm activity upon encapsulation within negative liposomes. Another work reports the efficacy of conventional but neutral liposomal antibiotic formulations. Moreover, the study investigated encapsulating the antibiotic cefoperazone within conventional liposomal formulations and reported a significant reduction of its biofilm inhibitory concentration by half [83].

As explained previously, one way to increase an agent’s selectivity and bioavailability is the concept of controlled release: the incorporation of constituents that only respond to certain internal or external stimuli [86]. Accordingly, various authors incorporated liposomal formulations with responsiveness to stimuli such as light, irradiation, and temperature, which proved efficacious against *P. aeruginosa* biofilms [67,69,85,87,88,89]. Photodynamic therapy involves the irradiation of a photosensitizer to generate toxic oxygen-reactive species, consequently killing bacteria [69]. Since biofilms are characterized by hypoxia, a method to carry oxygen into biofilms is required [69]. Accordingly, when the oxygen carrier, perfluorohexane was co-encapsulated with a photosensitizer, notable in vivo and in vitro antibiofilm activity were observed, and marked biofilm penetration and biofilm-associated hypoxia relief were achieved [69]. Similarly, in their study, Mai, et al., employed the photosensitizer DVDMS encapsulated within cationic liposomes constituting DOTAP:DOPE:cholesterol (0.31:0.46:0.23) in conjunction with photodynamic antimicrobial therapy [87]. In comparison with sole light exposure or unstimulated liposomal-DVDMS treatment, liposomal photos irradiated with light led to notable *P. aeruginosa* biofilm destruction in burn infection both in vitro and in vivo, with a notable safety profile [87]. Similar to photodynamic therapy, photothermal therapy entails the generation of lethal heat through the absorption of light, necessitating the presence of photothermal agents [67]. With their high packing density, phospholipid-porphyrin conjugates make excellent candidates for bimodal photothermal and photodynamic therapy due to their high photothermal conversion efficiency and their photodynamic activity [67]. When loaded with either of the photosensitizers pheophorbide-a and pyropheophorbide-a, significant antibiofilm activity was observed, with the former photosensitizer being the most effective, possibly due to its higher photodynamic efficiency [67].

As for the use of stimuli-responsive liposomes for enhancement of antibiotic efficacy against *P. aeruginosa* biofilms, studies are conflicting. As a therapeutic option exploiting the synergy between antibiotic administration and photothermal therapy, a group designed a repertoire of NIR-activated thermosensitive liposomes, encapsulating tobramycin and a NIR photothermal therapeutic agent [85]. The rationale behind liposomal encapsulation was to overcome the overlooked limitation of photothermal therapy: nonlocalized heat emission. Accordingly, liposomal formulations composed of different DSPC:cypate:BC ratios were synthesized (4:0.5:1, 4:0.5:2, and 4:0.5:3) denoted TSL1, TSL2, and TSL3, respectively [85]. Notably, tobramycin antibiofilm activity increased up to 8-fold upon NIR irradiation with TSL3, leading to 50% elimination at a dose of 15.6 μg/mL [85]. Moreover, TSL3 displayed the highest stability and tobramycin release, potentially owing to its higher BC content. However, opposing Zhao, et al., another paper discloses that liposomal encapsulation of tobramycin undermined its efficacy [85,89].

To simultaneously evade unwanted binding with the matrix and erode the biofilm’s integrity for deeper penetration, vapor nanobubbles generated from laser irradiation of light-responsive liposomes functionalized with either gold or graphene quantum dots were employed. Interestingly, while liposomal encapsulation enhanced tobramycin’s effect, electrostatic functionalization with gold led to premature leakage of the antibiotic. While graphene quantum dots functionalization led to stable liposomes with high encapsulation efficiency and no burst release, it failed to amplify tobramycin’s antibiofilm efficacy. Accordingly, laser-irradiation had no or even adverse effect on the antibiotic’s activity [89]. Using a metal prosthesis infection model, alternating magnetic fields were also used as exogenous stimuli in conjunction with temperature-sensitive liposomes bearing the antibiotic ciprofloxacin [88]. Alternating magnetic fields were used as a source of heat for biofilm disruption and the release of ciprofloxacin from the thermosensitive liposomes. While this encapsulation led to a 3-log reduction in colony-forming units of biofilm-bounded *P. aeruginosa*, this reduction was, in fact, not superior to that of the free antibiotic [88]. In light of these studies, more investigations of photodynamic therapy’s enhancement of antibiotic effects against biofilms should be executed.

Aside from stimuli-responsive formulations, various strategic formulations were employed to enhance the antibiotic’s effect [40,83,90,91]. As stated previously, hypoxia is a well-established property of biofilms. Specifically in cystic fibrosis patients, *P. aeruginosa* amplifies the expression of secreted phospholipase A_2_ (PLA_2_) and hypoxia in bronchial epithelial cells. Insightfully exploiting these extreme conditions, Rao, et al., created liposomal formulations that dissemble upon exposure to high levels of PLA_2_ [91]. These formulations encapsulated the antibiotic azithromycin, the antibiotic adjuvant 6-NIH that activates upon exposure to hypoxia after transforming to 6-AIH, and the biofilm dispersant: the nitric oxide donor: DETA NONOat. Remarkably, the formulations significantly eliminated mature *P. aeruginosa* biofilms, prevented *P. aeruginosa* adherence to airway epithelial cells, and, most importantly, killed azithromycin-resistant *P. aeruginosa* and eradicated their biofilms [91].

Another study was conducted using azithromycin and entailed encapsulating the antibiotic within nanoarchaeosomes. Nanoarchaeosomes are soft nanovesicles with a high resistance profile to degradation, such as enzymatic attacks. Comprised of biomaterials and polar lipids, of which some serve as ligands to scavenger receptor class AI (SRA1), they are innately targeted to cells expressing SRA1 [90]. This is of relevance as SRA1 play a pivotal role in innate immunity and cell apoptosis [92]. Notably, the group reveals that their inhalable nanoarchaeosomes composed of lipids extracted from the archaea *Halorubrum tebenquichense* displayed rapid biofilm penetration and structural stability than azithromycin [90]. Similarly, when encapsulated with nanoliposomes, meropenem was able to eradicate biofilm formation at doses significantly lower than that of the free antibiotic [40]. Of note, efficacy may vary depending on the strain.

In their work on *P. aeruginosa* strains PA01 and PA11, Gbian, et al., [93] reveal that the addition of PAβN, a broad-spectrum efflux pump inhibitor, enhanced the efficacy of liposome-encapsulated gentamicin and erythromycin against PA01 biofilm by at least two folds, but failed to do so for liposomal erythromycin against PA11. Interestingly, a recent work investigated the treatment of *P. aeruginosa* with liposomal perfluorohexane, an oxygen carrier, to relief biofilm-associated hypoxia, followed by treatment of free aztreonam, ceftazidime, or piperacillin-tazobactam [16] The work uncovered that this sequential treatment engendered markedly enhanced biofilm penetration, biofilm-associated hypoxia, and antibiotic efficiency against biofilm-bound bacterial cells [16]. Chitosan is an innately cationic biodegradable polymer with impressive biocompatibility, safety profile, and antimicrobial activity [94,95]. Interestingly, gentamicin encapsulated within phosphatidylcholine-chitosan nanoparticles and chlorhexidine encapsulated within chitosan hydrogels were far more efficacious against *P. aeruginosa* biofilms, especially in inhibiting biofilm formation in comparison with their free forms [94,95]. This potentially could be elucidated by chitosan’s positive charge, which permeates its interaction with the negatively charged biofilms [94,95].

Aside from antibiotics, unique liposomal formulations have been tested against *P. aeruginosa* biofilms. In addition, copper liposomal formulations were also incorporated in antibiofilm studies against *P. aeruginosa*, only to produce conflicting results. In one study, liposomes bearing copper nanoparticles proved inefficacious against *P. aeruginosa* biofilms [96]. However, another study [44] investigating copper liposomal formulations revealed that liposomes enclosing lipopeptide surfactant and copper nanoparticles led to reductions in preformed biofilms and biofilm formation prevention on urinary catheters, markedly superior to that of the free lipopeptide and the copper nanoparticle.

Given the importance of quorum-sensing systems in targeting biofilms, novel quorum-sensing inhibitors were synthesized and encapsulated within phosphatidylcholine/cholesterol liposomes, along with stearylamine. Due to their gradual release, enclosing these novel quorum-sensing inhibitors within liposomes along with the charged surfactant, stearylamine, led to a significant enhancement of antibiofilm activity [97]. In addition, human beta-defensin 2, a cationic antimicrobial peptide involved in innate immunity, has also been investigated for antibiofilm efficacy along with cholesteryl linoleate-containing liposomes [98]. Interestingly, this led to a marked alteration in biofilm cohesion, a decrease in biofilm mass, an increase in bacterial surface roughness, and an inhibition of extracellular structure emanation [98].

### 5.2. Escherichia coli Biofilms

*E. coli* is considered to be one of the most widespread bacteria, harboring acute resistance against antibiotic agents [99]. One study tested rhamnosomes, liposomes consisting of the antimicrobial surfactant rhamnolipid bearing the bacteriocin nisin Z against *E. coli* [100]. Similar to the results against *P. aeruginosa* biofilms, nanoliposomes were also markedly effective against *E. coli*, leading to a significant reduction in biofilm biomass and a disintegration in preformed biofilms [40]. Other groups also tested different liposomal formulations against *E. coli* biofilms, reporting significant efficacy [68]. Upon near-infrared irradiation, thermal liposomes bearing tungsten sulfide quantum dots and vancomycin managed to disrupt *E. coli* biofilms [68]. Another study also investigated stimuli-responsive liposomes and reported marked results. In their work, [101] investigated the antibiofilm efficacy of methylene blue encapsulated in various cationic liposomes made of different ratios DPPC:Chol:DOTAC. Interestingly, they report that methylene blue encapsulated in liposomes made from different lipid ratios of DPPC:Chol:DOTAC enhanced methylene blue biofilm penetration in comparison to its free form [101].

Liposomal azithromycin with and without N-acetylcysteine, an antioxidant, has been tested against *E. coli* biofilms [99,102] and both proved superior to their free form against *E. coli* SA057, yet not against *E. coli* SA10 biofilm. Moreover, liposomal azithromycin with N-acetylcysteine was comparable in efficacy to the free antibiotic, while liposomal azithromycin devoid of N-acetylcysteine was inferior to the antibiotic’s free form against *E. coli* SA057 [99]. Furthermore, another study tested azithromycin bound within propylene glycol (PGL-2), deformable propylene glycol (DPGL-2), or negatively charged liposomes with rigid bilayers (CL-3) [102]. Interestingly, while all liposomal formulations surpassed the efficacy of the free antibiotic, efficacy varied for each formulation and *E. coli* strain [102]. For instance, CL-3 was the most potent against *E. coli* ATCC 700928 and K-12 when it comes to biofilm formation prevention [102]. Finally, investigating the efficacy of liposomal formulations bearing tobramycin (TL) and Tobramycin-N-acetylcysteine (TNL) showed heightened antibiofilm activity of TL and TNL ensuing encapsulation against Tobramycin resistant *E. coli* [103].

### 5.3. Acinetobacter baumannii Biofilms

*A. baumannii* is a common culprit in nosocomial infections [71]. Nevertheless, commonly used first-line antibiotics are losing their efficacy against this bacterium [104]. Accordingly, several groups have tested liposomes against *A. baumannii* biofilms [71,103,104,105]. Notably, effective doses of TNL and TL were active against Tobramycin-resistant *A. baumannii* upon encapsulation of the drug [103]. Another study reports that a combination of chitosan-modified polymyxin B-loaded liposomes and ultrasound microbubbles almost completely eliminated biofilm-producing bacteria leading to results superior to free antibiotic or chitosan-modified polymyxin B-loaded liposomes [71]. However, ultrasound microbubbles with polymyxin B also managed to produce similar effects and almost eradicated biofilm-producing *A. baumannii* [71]. Furthermore, the light-emitting diode (LED)-activated liposomes enclosing silver sulfadiazine doped by curcumin produced marked reductions in bacterial number in comparison with sole LED exposure and other control groups [105]. Finally, *A. baumannii* biofilms have also been targeted by thymoquinone-bound liposomes, yet, both liposomal and free thymoquinone produced similar results [104].

### 5.4. Salmonella Species Biofilms

*Salmonella* is a common etiological agent behind foodborne enteric infections and is responsible for over 155,000 deaths globally per year [106]. Conventional liposomes bearing different compounds have been investigated against *Salmonella* biofilms [106,107]. One group investigated the efficacy of liposomal formulations bearing geraniol, a monoterpenic alcohol found naturally in various essential oils [106]. They report that liposomal geraniol inhibited the virulence of *Salmonella*’s serotype (*Salmonella typhimurium* ATCC 14028), reducing its colonization and adherence more effectively than the free drug [106]. In addition, the efficacy of other compounds found in essential oils were investigated against *Salmonella enterica* [107], including the two major terpenoids found in oregano and thyme: carvacrol and its isomer thymol. Interestingly, free carvacrol and free thymol were able to inactivate bacteria adhered to stainless steel after 1 min of contact; however, the liposomal formulations bearing a combination of both needed 10 min to inactivate the bacteria [107] significantly. Accordingly, more studies should be conducted against *Salmonella* species.

**Table 2:** Summary of liposomal drug delivery systems against gram-negative bacteria (not active (-); active (+); very active (++)).

Bacterial Strain	Liposome Type	Active Compound	Dosage	Efficiency	Refs.
*Pseudomonas* *aeruginosa*	Cationic and anionic with/out PEGylation	None	0–8 μmol/mL.	Anionic liposomes (-) Cationic liposomes (+) PEGylated cationic liposomes (++)	[81]
	Surface cationic lysozyme liposomes	Gentamicin	10 μL	Liposomal gentamicin with surface cationic lysozyme (++) Control (-)	[84]
	Cationic liposomes	Tobramycin + anti-biofilm peptide	≥4 and ≤32 μg/mL	Tobramycin enclosed in liposomes, with/out peptide (++)	[36]
	Neutral: NLG Negatively charged liposomes: NLG-1 and NLG-2.	Gentamicin	NLG: 0.125–0.5 mg/L.	*Pseudomonas aeruginosa*: NLG and NLG-1 (++) Control and NLG-2 (+) *K. oxytoca*: Control (-) NLG (++) NELG-1, and NELG-2 (+)	[78]
	Cationic Liposomes + photodynamic therapy	DVDMS	10 μg/mL of DVDMS	Cationic lipid-mediated nano-DVDMS with Photodynamic therapy (++) Control (-)	[87]
	Photodynamic liposome	Perfluorohexane and photosensitizer	100 μL	Photodynamic liposomal mixture (++)	[69]
	Bimodal photodynamic and photothermal liposomes	Phospholipid-porphyrin conjugates + pheophorbide-a (Ph_x_LPC)/pyropheophorbide-a (Pyr_x_LPC)	Ph_3_LPC:1.63 μM Pyr_3_LPC: 5.45 μM	Ph_3_LPC (++) Pyr_3_LPC (+)	[67]
	Thermosensitive liposomes of varying DSPC: cypate: Betainylated Cholesterol ratios TSL1: (4:0.5:1) TSL2: (4:0.5:2) TSL3: (4:0.5:3)	Tobramycin + cyanine dye	15.6 ug/mL	TSL1 (+) TSL2 (+) TSL3 (++)	[85]
	Light responsive liposomes	Tobramycin + gold nanoparticles (Lip-AuNP) + graphene quantum dots (GQD)	16 µg mL^−1^	Tobramycin with Lip-AuNP or GQD (-)	[89]
	Temperature-sensitive liposomes + alternating magnetic fields	Ciprofloxacin	MIC = 0.5 µg/mL	Free/liposomal ciprofloxacin (+)	[88]
	Nanoarchaeosome	Azithromycin (AZ)	8 μg/mL	AZ-nanovesicles/nanoarchaeosome (++) Free AZ (-)	[90]
	Neutral liposomes	Meropenem	6.25 µg/mL	Liposomal meropenem (++) Control (+)	[40]
	Adjuvant liposomes	Azithromycin (AZI) + (2-nitroimidazole derivative, 6-NIH) + (nitric oxide donor, DETA NONOate).	120 μg/mL	Liposomal mixture (++) Control (+)	[91]
	Neutral liposomes	Cefoperazone sodium	IC50: 0.42 μg/mL	Liposomal cefoperazone (++) Control (+)	[83]
	Neutral liposomes	Gentamicin (GEN) or erythromycin (ERY) + PABN	Liposomal GEN with PABN: 1–8 mg/mL Liposomal ERY with PABN: 64–128 mg/mL	Against PA01: PABNA with liposomal GEN/ERY (++) Against PA11: PABNA with liposomal GEN (++) PABNA with liposomal ERY (+)	[93]
	Proliposomes	Tobramycin + clarithromycin (TOB/CLA-CPROLips)	0.06 to 16 mg/L	TOB/CLA (-) CPROLips (++) Control (+)	[108]
	Neutral liposomes	Perfluorohexane followed by aztreonam	100 μL	Sequential treatment (++) Control (-)	[16]
	Neutral liposomes	Chitosan + phosphatidylcholine + gentamicin	10 μL of liposomes loaded with 20 μg/mL gentamicin	Liposomal mixture (++) Control (+)	[95]
	Neutral liposomes in Chitosan hydrogels.	Chlorhexidine	100 μL	Chitosan hydrogels loaded with liposomal chlorhexidine (++) Control (+)	[94]
	Neutral liposomes	Copper	-	Liposomes loaded with copper nanoparticles (-)	[96]
	Neutral liposomes	Lipopeptide surfactant + copper nanoparticles	89 μg/mL	Liposomes enclosing lipopeptide surfactant with copper nanoparticles (+) Control (+)	[44]
	Phosphatidylcholine/cholesterol liposomes with stearylamine: (LP-SA/CDC) and (LP-SA/PF)	Novel quorum sensing inhibitors: “CDC” and “PF”	1–10 µM	LP-SA/CDC (+) LP-SA/PF (++)	[97]
	Liposomes made of cholesteryl linoleate (CL-PL)	human beta-defensin 2 (HBD2)	CL:125 µg/mL HBD2: 0.25 µM.	CL-PL with HBD2 (++) Control (+)	[98]
	Neutral liposomes	Commercial ozonated sunflower oil + hypromellose	Final concentration of 20% of the ozonated sunflower oil	Liposomal formulations (++)	[109]
*Escherichia coli* *Pseudomonas aeruginosa*	Rhamnosome nano-vesicles	Nisin Z	*Escherichia coli*: 25 μg/mL *Pseudomonas aeruginosa* 100 μg/mL	Nisin Z encapsulating rhamnosomes (++) Control (+)	[100]
*Escherichia coli*	Thermal Liposomes	Tungsten sulfide quantum dots (WS2QDs) and vancomycin	50 μg/mL	Liposomal formulations with NIR (++) Control (-)	[68]
	Cationic liposomes made of DPPC:Chol:DOTAC. Lip1 (1:0.5:0.3) Lip 2 (1:0.5:0.5) Lip 3 (1:0.5:0.8) Lip 4 (1:0.5:1.2)	Methylene blue	Final molar concentration of MB of 0.02% *w*/*v*	Lip3, Lip2, Lip1 (+) Lip4, Control (-)	[101]
	Neutral liposomes	Azithromycin (LA) or azithromycin/N-acetylcysteine (LAN)	2.5–3 μg/mL	Strain SA057: LA and LAN (++) Free drugs+ Strain SA10: LA (+) LAN and Control (++)	[99]
	Negative liposomes with rigid bilayers (CL-3), propylene glycol liposomes (PGL-2), or deformable propylene glycol liposomes (DPGL-2)	Azithromycin (AZI)	Varied among strains and liposomal formulations within 1.26–45.65 µg/mL	DPGL-2 (++) CL-3 and PGL-2 (+)	[102]
	Solid liposomes	Clove oil	0.5 mg/mL	Liposomal formulations (+)	[110]
	Functionalized PEG-liposomes.	MTAB	In 3.6 µmol of lipids, 67.5 µg/mL of attached MTAB	Liposomal MTAB (++) Free MTAB (+)	[111]
*Acinetobacter baumannii*	Chitosan-modified liposomes (CLPs) + ultrasound microbubbles (USMBs)	Polymyxin B	8 ± 2 µg/mL	USMBs with CLPs (++) Control (+)	[71]
	Silver sulfadiazine liposomes (AgSD-NLs@Cur)	Curcumin	7.8 μg/mL	LED plus AgSD-NLs@Cur (++) Control (-)	[105]
	Neutral liposomes	Thymoquinone (TQ)	TQ: 2 and Lip-TQ: 4 µg/mL	Control (++) Lip-TQ (+)	[104]
*Acinetobacter baumanii Escherichia coli* *Klebsiella pneumoniae*	Neutral liposomes	Tobramycin or tobramycin-N-acetylcysteine	Varying across strain and formulation within 8 to 128 mg/L.	Control (+) TNL and TL (++)	[103]
*Salmonella enterica*	Neutral liposomes	Thymol and carvacrol	0.662 mg/mL	Control (++) TCL (+)	[107]
*Salmonella typhimurium*	Neutral liposomes	Geraniol	0.10%	Geraniol-Loaded Liposomal (+)	[106]
*Aeromonas hydrophila* *Serratia grimesii*	Neutral liposomes	Curcumin	0.35 mg/mL	Curcumin liposomes (++)	[112]
*Aeromonas sobria*	Neutral liposomes	Curcumin	420 µg/mL	Control and liposomal curcumin (++)	[113]
*Porphyromonas gingivalis*	Liposomes consisting of quaternary ammonium chitosan (TMC)	Doxycycline (DOX)	1 mL	TMC-lip-DOX (++) Control (+)	[114]
*Porphyromonas gingivalis* and *Prevotella intermedia* mixed biofilm	Liposomes (TMC-Lip-DOX NPs) composed of TMC	Doxycycline	1 mL	TMC-Lip-DOX NPs (++) Control (+)	[115]

## 6. Liposomal Formulations for the Treatment of Gram-Positive Biofilms

As with gram-negative bacteria, infections due to gram-positive biofilms present a major health challenge, especially due to the emerging resistance of gram-positive strains to antibiotics [116,117]. Gram-positive bacteria are responsible for the majority of infections in intensive care units in hospitals and are the leading cause of skin, soft tissue, and medical device-associated infections [118,119]. Further adding to the urgency of gram-positive medical infections is their susceptibility to form biofilms, which are generally responsible for the vast majority of clinical chronic infection cases [120,121]. Several types of clinically relevant gram-positive strains that have the tendency to develop biofilms include the *Staphylococcus* strains: *S. aureus,* including *Methicillin-resistant S. aureus* (MRSA) and *Methicillin-susceptible Staphylococcus aureus* (MSSA); *S. epidermidis*; and *Staphylococcus saprophyticus *subspecies* Bovis*, as well as other gram-positive strains, such as *Listeria*, *Mycobacterium*, *Cutibacterium acnes* (*C. acnes*), and *Bacillus subtilis*. Various liposomal composites have been developed in efforts to overcome the resistance and treat biofilms of those bacterial strains. The subsequent sections discuss the progress in the use of liposomal formulations for the treatment of gram-positive biofilms. Specific focus is given to therapeutic parameters, including triggered release, biofilm targeting mechanisms, loaded drugs, including antibiotics and their alternatives, and the efficiency of the composite at eradicating the biofilm in vitro and in vivo. Table 3 summarizes all the studies investigating liposomal delivery systems for gram-positive anti-biofilm activity.

### 6.1. Staphylococcus aureus Biofilms

*S. aureus* is responsible for the most commonly encountered bacterial infection cases among hospitalized patients, and results in significant mortality despite the administration of therapeutics. The problem of *S. aureus* infections is further intensified by the rising prevalence of drug-resistant strains of the bacteria and their development into biofilms. Of special clinical relevance is the MRSA strain, which is responsible for the majority of morbidity and mortality cases [122,123,124,125]. MRSA has the ability to populate and infect skin, wounds, and the circulatory system. Treatment of MRSA is greatly hindered by the development of resistance to antibiotics, such as daptomycin and vancomycin [126]. Although several drugs show promise for MRSA eradication, those drugs are generally limited by factors including low water solubility, stability, and bioavailability [35,126].

Furthermore, *S. aureus* is responsible for the majority of primary skin infections, with one of the challenges of its treatment being resistant strains such as MRSA and biofilm development. The main treatment strategy for such skin infections involves the topical application of antibiotics. However, this introduces another set of treatment challenges, including insufficient local drug concentration, increased antibiotic-resistant strains, biofilm formation, or inability of the drug to reach the site of action, which necessitates the administration of high antibiotic doses via oral or parenteral routes. This, in turn, raises the risks of developing antibiotic resistance and experiencing side effects and allergic reactions [34].

Therefore, liposomal formulations of antimicrobial drugs were developed as suitable drug delivery systems to overcome those limitations. Platensimycin, for instance, is a potent antibiotic against gram-positive bacteria, including MRSA. However, the clinical utilization of platensimycin has been impeded due to its low solubility and poor pharmacokinetics [126]. Wang, et al. [126] investigated the encapsulation of platensimycin within bare and mannose-modified liposomes to improve the drug’s pharmacokinetic profile and assess its potential to eradicate MRSA and MRSA biofilms. The liposomal platensimycin formulation effectively inhibited the growth of MRSA in vitro to a degree comparable to that of unencapsulated platensimycin, even at low concentrations. This is in contrast to other antibiotics, such as vancomycin. In vivo, the liposomal formulations were capable of increasing survival in MRSA-infected mice by 20 to 40%, although the mice still suffered from infection-related glomerular injury, inflammatory cell infiltration, and edema (interstitial and intracellular) of the kidneys.

Cationic liposomes were also investigated to improve the delivery of poorly soluble anti-biofilm agents. Aiello, et al. [35] investigated the use of glycosylated cationic liposomes for the MRSA-specific delivery of the drug trans-resveratrol (trans-RSV) for biofilm elimination. RSV is a drug that inhibits the growth of gram-positive and -negative biofilms by inhibiting their Quorum sensing system through which they communicate with other bacterial cells within the biofilm. Despite its promising anti-biofilm activity, low price, and good safety profile, RSV is limited by its low water solubility and hence, bioavailability, as well as its instability in physiological fluids. In this study, ammonium-modified glycosyl moieties were incorporated into the RSV-loaded liposomes to target MRSA biofilms via electrostatic interaction and specific binding between the liposome and the bacteria-/biofilm-overexpressed carbohydrates. Although mannose, galactose-, and glucose-modified liposomes investigated in this study entrapped RSV with higher efficiency than unglycosylated liposomes, their anti-biofilm activity varied. Galactosylated RSV-loaded liposomes exhibited good anti-biofilm activity against preformed MRSA biofilms, followed by mannosylated RSV liposomes, while glucosylated liposomes did not demonstrate an anti-biofilm effect. Those results indicate galactosyl groups can serve as efficient MRSA biofilm targeting moieties for drug delivery systems such as liposomes. Furthermore, this study avoids the use of antibiotics hence eliminating the possibility of antibiotic resistance by targeting the Quorum sensing system to demolish the biofilm [35].

Cationic liposomes were also studied with surfactants for the elimination of MRSA biofilms. Surfactants are amphiphilic compounds of natural or synthetic origins that disintegrate biofilms and prevent their formation [127]. Lin, et al. [32] used nano-sized liposomes intercalated with the amphiphile (surfactant) SME for anti-MRSA and -MRSA biofilm activity. This study reported the ability of the liposome-surfactant formulation to reduce biofilm thickness to an extent equivalent to that of antibiotic ciprofloxacin and amphiphile cetylpyridinium chloride. Furthermore, in an in vivo MRSA skin-infected mouse model, the SME-incorporated liposomes induced a notable reduction in the wound’s infection, although edema was still apparent at the infection site [32].

Treatment of MRSA skin infections using liposomal antibiotic formulations was also explored by Rukavina, et al. [34]. In this study, the orally, parenterally, and ophthalmically administered antibiotic azithromycin was incorporated into different liposome types and studied for potential topical applications for the treatment of MRSA skin infections. This study investigated AZT loaded into neutral liposomes (CLs), cationic liposomes (CATLs), deformable liposomes (DLs), and propylene glycol-incorporated liposomes (PGLs) for topical azithromycin applications. While CLs and CATLs had rigid membranes, DLs and PGLs were elastic, with DLs having the highest membrane elasticity. All the studied liposomes efficiently encapsulated and released azithromycin with the higher efficiencies reported for the elastic liposomal formulations. In terms of activity against MRSA, all liposomal azithromycin formulations had significantly higher activity than free azithromycin against both MRSA planktonic bacterial cells and biofilm, with the highest activity reported for CATLs. The high antibiofilm activity of CATLs is possibly due to their positive surface charge, which can facilitate their interaction with the negatively charged biofilm surface, as well as the presence of the cationic lipid DODAB reported to have antimicrobial activity. Therefore, this study provides valuable information on the effect of varying the composition, surface charge, and elasticity on the release behavior and interaction of the liposomes with bacteria, whether planktonic or biofilms [34].

Liposomes have been used to load other drugs restricted by their low water solubility, bioavailability, and cell penetration ability, such as the bactericidal drugs curcumin and berberine. Liposomes have been used for the dual loading of curcumin (within the lipid bilayer) and berberine (in the aqueous interior) for the elimination of MRSA biofilms in vitro. Although dual curcumin/berberine treatment had no anti-MRSA effects, co-loading them within liposomes significantly reduced the minimum inhibitory concentrations (MICs) of the drugs due to their synergistic antimicrobial activity. Therefore, the co-loaded liposomes surpassed the free drugs in inhibiting impeding MRSA growth and hampered biofilm formation in vitro. Furthermore, the curcumin/berberine-loaded liposomes were biocompatible in vitro [128].

Scriboni, et al. [39] studied different liposomal formulations to carry the drug vancomycin for MRSA and MSSA biofilm eradication in vivo. The types of liposomes encapsulating vancomycin studied were conventional large unilamellar vesicles, fusogenic unilamellar vesicles, and cationic unilamellar vesicles. Although there was no difference in MRSA and MSSA biofilm inhibition between the vancomycin-loaded liposomal formulation and the free drug, the fusogenic liposomal vancomycin eliminated mature biofilms to a degree surpassing that of other liposomal formulations as well as the free drug [39].

Nanoparticles have been explored as promising antibiotic alternatives to fight multidrug-resistant bacterial strains, including MRSA. Although promising as antibacterial agents, some nanoparticles suffer from issues such as toxicity, thereby requiring the use of a nanoencapsulation delivery system such as liposomes [44,129]. Kannan, et al. [44] studied multilamellar liposomes encapsulating copper nanoparticles and the biosurfactant lipopeptide for antibiofilm activity [44]. In this study, liposomes loaded with lipopeptide produced from *Paenibacillus thiaminolyticus* and copper nanoparticles (CuNPs) significantly reduced the minimum inhibitory concentration of MRSA compared to CuNP or lipopeptide, and a very fast anti-MRSA bactericidal activity via the intracellular accumulation of ROS and membrane damage. In terms of antibiofilm activity, the lipopeptide/CuNP liposomes significantly reduced biofilm, indicating their strong antibiofilm activity. Furthermore, the loaded liposomes reduced biofilm thickness and adherence within the biofilm and showed some inhibition of extra polymeric matrix secretion. The antibiofilm effect of the loaded liposomes was also evident in vitro when coated onto urinary catheters [44].

Based on our search, no subsequent studies investigated the use of nanoparticle-loaded liposomes for MRSA antibiofilm effects. However, other biosurfactants have been studied with liposomes for MRSA biofilm reduction. Biosurfactants are bactericidal agents generated by microorganisms living in a competitive environment and have been studied as safe antimicrobial and antibiofilm agents [130]. Giordani, et al. [131] studied the incorporation of a nontoxic biosurfactant extracted from Lactobacillus gasseri into liposomes and reported a significantly improved ability to prevent the formation of and eliminate MRSA biofilms compared to the bare biosurfactant [131].

Internal and external stimuli-responsive liposomes have also been briefly explored for MRSA-targeted antibiofilm effect [72,132]. Low pH-responsive multilamellar liposomes composed of the charged oleic acid (anionic) and stearyl amine (cationic) lipids stacked into layers via electrostatic interactions have been studied for MRSA-specific delivery of the hydrophilic antibiotic vancomycin. Response to the low pH characteristic of bacterial environments is induced by protonation of the oleic acid and stearyl amine complexes in acidic environments leading to repulsion between the lipids, subsequent destabilization of the liposomes, and release of encapsulated cargo. The results of this study showed the synthesis of biocompatible onion-like multilamellar vesicles with a high vancomycin entrapment efficiency with a faster drug release at low pH. Although drug release from the liposomes was faster at low pH, the total amount of released drug at both pHs was very similar. However, the trapping of vancomycin within multilamellar vesicles reduced the MIC of vancomycin by 8-fold at both physiological and acidic pHs.

In terms of antibiofilm activity, multilamellar vesicles loaded with vancomycin-induced MRSA antibiofilm activity compared to unencapsulated vancomycin. In vivo evaluation of the activity of vancomycin-loaded multilamellar vesicles in mouse models with MRSA skin infections revealed a notable increase in bactericidal activity of the encapsulated composites, compared to the bare drug as indicated by the significantly reduced colony finding units. The improved antibacterial effects reported in this study could stem from two factors. The first factor is the charge change from negative to positive at low pH, which can improve the electrostatic attraction with the bacteria. The second factor is the pH-triggered release resulting in higher vancomycin concentrations at the target site [132].

The extrinsic stimuli, the ultrasound, has been reported to trigger release for MRSA antibiofilm activity by Zhou, et al. [72]. The antibiotic peptide human beta-defensins-3 (HBD-3) liposome microbubbles were exposed to low-frequency ultrasound to achieve targeted biofilm diagnosis and treatment. Liposome microbubbles can protect HBD-3 from degradation in vivo and maintain its high concentration. This study reported that combining low-frequency ultrasound with HBD-3 liposome microbubbles significantly boosts the bactericidal effects of HBD-3 against MRSA biofilms and prevents further development of the biofilm in mouse models [72].

Another *S. aureus* strain responsible for much mortality is MSSA, which is responsible for between 50% and 99% of staphylococcus infections. The issue of treating MSSA infections is further magnified by its ability to develop into a biofilm that is resistant to antibiotics. The primary treatment for MSSA infections typically involves the administration of the drug nafcillin. PEGylated liposomes were used to encapsulate nafcillin and, compared to conventional liposomes encapsulating nafcillin, the stealth liposomes raised the carrier’s drug loading efficiencies and increased its release period [133]. Furthermore, coating with PEG reduced the MIC by 2-fold, as compared to the unmodified liposomal nafcillin. It is expected that this reduction in MIC is due to the greater positive surface charge of the PEG-modified formulations allowing a better interaction with the negative surface charge of the bacteria. The release profile of the loaded stealth liposomes resulted in reduced nafcillin toxicity towards human cells. In terms of anti-biofilm activity, entrapping nafcillin into PEGlyated liposomes improved the drug’s anti-biofilm activity by 8-fold compared to the bare drug. This anti-biofilm effect of drug-loaded stealth liposomes surpassed that of the un-PEGylated formulation by two folds. Although this study showed improved performance and safety of PEGylated nafcillin liposomes compared to liposomal nafcillin and bare nafcillin in animal models, it does not assess the anti-biofilm effect of the composite in animal models [133].

Ferreira, et al. [134] studied the loading of the broad-spectrum antibiotic rifabutin into liposomes of varying compositions against planktonic and MSSA biofilms. Rifabutin was loaded into neutral, positive, and negative liposomes, fusogenic liposomes, and different transition-temperature (−16, +23, +41 °C) liposomes. This study reported that all rifabutin-loaded liposomes preserved rifabutin’s antibacterial, although MIC varied slightly between the different compositions. In addition, negatively charged liposomes showed lower minimum biofilm inhibitory concentration (MBIC) and a higher percentage in biofilm reduction, while also preserving the cell viability of normal cells in vitro, unlike their positively surface-charged counterparts [134].

Several other studies evaluated liposomal formulations to encapsulate and deliver anti-biofilm agents, including antibiotics and nanoparticles, for the treatment of *S. aureus* biofilms. Azithromycin’s antibacterial activity is restricted by its administration via oral and parenteral routes, which limits its ability to access the biofilm or be retained within it at concentrations high enough to have a therapeutic effect. Altube, et al. [90] reported the first inhalable azithromycin liposomal formulation for *S. aureus* biofilm eradication. The liposomes are composed of total polar archaeolipids containing PGP-me extracted from Halorubrum tebenquichense archaebacteria, which equips the formulation with the ability to target the scavenger receptor class AI [90]. The scavenger receptor is an important part of the alveolar macrophage-mediated immune response in lung infections in which they bind to gram-positive and -negative bacteria to eliminate the bacteria [135]. The targeted and drug-encapsulated liposomal formulation did not have a bactericidal activity higher than that of the bare drug against S. aureus and failed to disrupt its biofilm in vitro [90].

However, several other studies reported improved biofilm eradication by antibiotic-liposomal formulations. For example, cefquinome sulfate encapsulated within cationic liposomes achieved a biofilm annihilation twice that of the free drug [136]. In contrast, dual levofloxacin- and serratiopeptidase-loaded liposomes eliminated the majority of the preformed biofilm in vitro [137]. Lysozymes, proteases that cleave polysaccharides in bacterial pathogens [138], have also been explored with liposomes to improve the antibiofilm effects of antibiotics [84,139]. Anionic liposomes have been electrostatically associated with the positively charged lysozymes and loaded with the antibiotic gentamicin to yield cationic gentamicin liposomes, as reported by Hou, et al., [84]. As previously discussed, the positive surface charge of liposomes is advantageous due to its improved interaction with the negatively charged bacterial and biofilm surfaces [34]. This study showed that lysozymes stabilized the liposomes, preventing the leakage of gentamicin from the liposomes.

In terms of anti-biofilm effects, the lysozyme-modified liposomes encapsulating gentamicin had an anti-biofilm activity surpassing that of the individual drug or lysozyme and prevented the biofilm development from planktonic cells [84]. The observed prevention of biofilm formation could be due to the reduced bacterial adhesions resulting from the attractive electrostatic forces between the cationic liposomes and the negatively charged biofilm surface [15,44,45]. Lysozyme-modified liposomes were also explored with the drug levofloxacin for S. aureus biofilm eradication. This study reported the high entrapment efficiency of levofloxacin within liposomes and its controlled release as well, and suggested this could indicate prolonged anti-biofilm effects [139].

To improve the performance of the antibiotic-loaded liposomes in *S. aureus* biofilm eradication, targeted liposomes specifically for *S. aureus* biofilms have been developed [59,60,67,68,96,140,141]. The liposomes are conjugated to α-hemolysin antibodies and loaded with isosorbide mononitrate. α-hemolysin is a protein involved in the development of *S. aureus* biofilms, while isosorbide mononitrate is a NO donor that benefits from NO’s antimicrobial and anti-*S. aureus* biofilm abilities to treat infections. These loaded immunoliposomes had a stronger inhibitory effect on the formation of biofilms than isosorbide mononitrate untargeted liposomes or single isosorbide mononitrate. At the highest concentration of 45 mg/mL, the loaded immunoliposomes had the highest inhibitory effect on the formed biofilms compared to loaded liposomes (untargeted) and the bare drug at 11 mg/mL. It is worth noting that the drug-loaded liposomes performed better than the bare drug with formed and forming biofilms [59].

Aptamers are other good targeting agents due to their ability to bind to their targets with high affinity. Ommen, et al. [60] reported that the thermoresponsive aptamer-modified targeted liposomes accumulated within and fully penetrated the *S. aureus* biofilm. The in vitro study showed that mild hyperthermia triggered the release of vancomycin and rifampicin from the thermoresponsive aptamer-conjugated liposomes, and annihilated the *S. aureus* biofilm. Although only the aptamer-conjugated liposomes could kill all bacterial cells within the biofilm, the modification with an aptamer did not improve the formulation’s antibacterial effects [60].

Temperature-responsive liposomes were studied to load nanozymes and antibiotics for *S. aureus* biofilm elimination. Nanozymes are nanomaterials that perform enzymatic activities, such as mimicking the activity of the enzyme peroxidase by converting hydrogen peroxide (H_2_O_2_) into free radicals that destroy bacterial cells (chemodynamic therapy). However, the efficiency of chemodynamic therapy in the elimination of bacteria is usually reduced due to the high glutathione (GSH) levels typically present in sites of infection. Therefore, Xu, et al. [68] explored heat-responsive liposomes to encapsulate the antibiotic vancomycin and peroxidase-like nanozymes active at neutral pH and possessing oxidase activity to oxidize GSH and improve chemodynamic therapy. Tungsten sulfide quantum dots (WS_2_QDs) were used as peroxidase- and oxidase-like nanozymes, which are also capable of heat generation upon exposure to near-infrared light (photothermal conversion). This heat induces a phase transition of the hyperthermia-responsive liposomes and release of the encapsulated vancomycin, resulting in synergistic bactericidal activity of the drug and the chemodynamic therapy. This formulation eradicated the vancomycin-intermediate *S. aureus* strain (Mu50) and possibly facilitated vancomycin’s penetration into the biofilm. In vivo, the WS_2_QD/vancomycin-loaded liposomes retained antibacterial activity while also maintaining a good safety profile [68].

Photothermal therapy (PTT) has been explored in combination with photodynamic therapy (PDT), where a photothermal material absorbs light and converts it to heat that can kill the bacteria, while a photosensitizer generates bacteria-toxic reactive oxygen species from oxygen. Dual PTT/PDT for *S. aureus* biofilm eradication was studied using liposome-like assemblies composed of organic phospholipid-porphyrin conjugates. The bimodal liposome-like assemblies showed anti-planktonic and -biofilm activity, particularly upon application of NIR irradiation in vitro [67].

Dual PDT/PTT was used for anti-*S. aureus* biofilm performance using gold-coated and curcumin-encapsulated incorporated liposomes. Curcumin is capable of photodynamic therapy when exposed to blue light, while gold nanoparticles exhibit photodynamic effects when exposed to NIR light. Curcumin/gold nanoparticle liposomes achieved a complete inhibition in the growth of the bacteria, and significantly prevented biofilm formation due to PTT generated upon NIR exposure and subsequent application of blue light. In addition, it is important to note that the gold nanoparticles coating the liposome had a positive charge which facilitated the composite’s adhesion to the negatively surface-charged biofilms, thereby further improving their activity against biofilms [140].

Single PDT using photosensitizer-encapsulated liposomes for activity against *S. aureus* biofilms was studied by Rout, et al. [142], who used the photosensitizer Toluidine Blue O and reported desirable anti-biofilm effects. Antibiotic alternatives such as bactericidal ozonated sunflower oil with hypromellose (together commercially known as Ozodrop^®^) have eradicated and prevented the formation of biofilms [109].

As with the external NIR stimulus, pH has been explored as an endogenous stimulus for the specific delivery of anti-*Staphylococcal* endolysin LysRODI that possesses lytic activity against *S. aureus* biofilms. Portilla, et al. [141] reported that these pH-responsive endolysin-loaded have antimicrobial activity against planktonic cells comparable to that of the free endolysin and a significant anti-biofilm activity against *S. aureus* 15981, V329, and Sa9 strains, yet to a degree slightly lower than that of free LysRODI [141].

Another versatile strategy compared to the previously described strategies are the studies by Raj, et al. [143] and Hemmingsen, et al. [94], which hybridized liposomes with the polymer chitosan to improve biofilm elimination. Raj, et al. [143] used the natural *S. aureus* antibiofilm agent alizarin, which is restricted due to its low solubility. As a result, alizarin liposomes coated with the polymers chitosan and gum arabic eliminated *S. aureus* biofilms to an extent surpassing that of alizarin drug-free carriers and reduced the MIC compared to the bare drug [143]. Furthermore, loading the liposomes with chlorhexidine and encapsulating it within chitosan hydrogel provided a synergistic effect and exhibited a sustained chlorhexidine release, improved chlorhexidine antibacterial activity compared to the free drug, and inhibition of biofilm formation of *S. aureus*, compared to free chlorhexidine in vitro [94]. However, based on our search, no further studies were conducted combining liposomes with other carriers, such as polymeric chitosan, for *S. aureus* biofilm treatment.

### 6.2. Staphylococcus epidermis

In addition to *S. aureus*, *S. epidermis* is an important pathogenic strain associated with medical implants, whose elimination is highly challenging [144,145]. Although liposomal formulations have been extensively studied for *S. aureus* biofilm treatment, it has only been briefly investigated for *S. epidermis* biofilms. Eroglu, et al. [145] used liposomes for the dual delivery of tetracycline and tretinoin to benefit from the two drug’s combinatorial effects at the disruption of *S. epidermis* biofilms. For skin applications, the liposomes lack the required viscosity; therefore, the liposomal formulation was incorporated into hydrogels. Lipophilic tretinoin and hydrophilic tetracycline HCl were released in a controlled manner from the liposome-hydrogel formulation compared to the hydrogel-only and liposome-only formulations, although an initial burst release was reported. In terms of anti-biofilm activity, the loaded hydrogel/liposomal composition reduced the MBIC compared to loaded hydrogel formulations, although the minimum biofilm eradication concentration remained unchanged between the two groups. However, the minimum biofilm eradication concentrations of both compositions were effective against biofilms compared to the unloaded hydrogel formulations [145].

Rifampin is an antibiotic with reported *S. epidermis* anti-biofilm efficacy, yet, the development of resistance against single-agent rifampin is expected. Therefore, Bazrgari, et al. [144] combined rifampin with N-acetylcysteine, a bactericidal non-antibiotic agent, within a liposomal nanocarrier for their delivery to biofilms. Unlike the free drug or dual drug treatment, which failed at eliminating the *S. epidermis* biofilms, the positively surface-charged liposomes loaded with drugs significantly eliminated formed biofilms. However, the liposomal formulations with N-acetylcysteine or combined N-acetylcysteine and rifampin were ineffective in the prevention of biofilm formulation, while rifampin effectively biofilm development. N-acetylcysteine’s effects, on the other hand, were dose-dependent. The antibiofilm effects of rifampin and liposomal rifampin did not differ significantly. Therefore, since no synergy was observed between N-acetylcysteine and rifampin in liposomal formulations, liposomal rifampin is the most promising formulation against *S. epidermis* biofilms in vitro [144].

Natsaridis, et al. [146] used liposomes for delivery of the antibiotic moxifloxacin against *S. epidermis* biofilms in vitro. However, unlike the previous studies reported in this paper, this study focused on the effect of the preparation of moxifloxacin liposomes on the formulation’s antimicrobial effects. Compared to the dehydration-rehydration preparation method, the active loading method allowed higher moxifloxacin encapsulation, longer moxifloxacin retention time, and significantly amplified biofilm inhibition and reduction. Unlike liposomal moxifloxacin, the free drug had a poor effect on formed biofilms [146].

Another study using a rather unique strategy compared to the previously discussed approaches involves the use of liposomal formulations of antibiotics together with agents that could disperse formed biofilms (biofilm dispersal agents) to fight biofilms, including *S. epidermis* biofilms. Liposomes have been reported for the delivery of the antibiotic vancomycin and the dispersal agent cis-2-decenoic acid to *S. epidermis* biofilms. This formulation exhibited a notable anti-biofilm activity surpassing that of the encapsulated therapeutic agents individually and combined. The anti-biofilm effects were reported to have concentration and time dependencies in vitro [147]. Similar to liposomal formulations against *S. aureus*, antibiotic alternatives have been studied with liposomes to combat *S. aureus* biofilms. The natural, poorly water-soluble antibacterial usnic acid has been delivered using glucosylated liposomes to *S. epidermis* biofilms. The use of glucosyl is advantageous as it could improve the targeting of the liposomal formulation to the bacterial cells via adhesion to bacterial lectins. Usnic acid-loaded glucosylated cationic liposomes had strong anti-*S. epidermis* biofilm activity, unlike bare usnic acid, which was even more pronounced below MIC. This improved performance can be attributed to the formulation’s surface positive charge and glycosidic groups improving the interaction between the liposomal composition and the biofilm [148]. Although the studies using liposomal vehicles against *S. epidermis* biofilms are rather few and conducted in vitro, the provided results support further investigations of these compositions for biofilm elimination of *S. epidermis*.

### 6.3. Staphylococcus saprophyticus *subsp.* Bovis Biofilms

Another even less studied *Staphylococcus* strain is *Staphylococcus saprophyticus* subspecies *Bovis*. Photodynamic therapy was explored as an alternative to antibiotics to coat catheters for *Staphylococcus saprophyticus* subspecies *Bovis* anti-biofilm performance. Vögeling, et al. [149] used liposomes loaded with a water-soluble complex of the hypericin, a potent lipophilic photosensitizer, and (2-hydroxypropyl)-β-cyclodextrin (Hyp-HPβCD) for anti-biofilm activity in vitro. This study further combines Photodynamic therapy with ultrasound to improve the biofilm penetration of Hyp-HPβCD. Dual light-induced Photodynamic therapy and ultrasound reduced biofilm growth to a greater extent than lone photodynamic therapy while maintaining biocompatibility in vitro [149]. The same group investigated liposomal Hyp-HPβCD for lone photodynamic therapy against *Staphylococcus saprophyticus* subspecies *Bovis*. Although the liposomal formulation reduced biofilm growth, the highest anti-biofilm activity was observed for free Hyp-HPβCD in vitro [150]. No further studies were conducted with liposomal formulations for *Staphylococcus saprophyticus* subspecies *Bovis* anti-biofilm activity. As with *S. epidermis*, the available anti-*Staphylococcus saprophyticus* subspecies *Bovis* biofilm liposomal formulations were limited to in vitro studies.

### 6.4. Streptococci Species

In addition to *Staphylococcus* bacterial strains, several other medically relevant bacteria have the tendency to form biofilms. However, liposomal compositions to treat biofilms belonging to those strains have not been as extensively studied. *Streptococcus pneumonia* (*S. pneumonia*), ranked by the world health organization as a priority pathogen, is responsible for a wide range of infections such as otitis media, meningitis, and sepsis. The treatment of *S. pneumonia* is challenged by its increased antibiotic resistance [151]. Furthermore, the formation of *S. pneumonia* biofilms further challenges its treatment [152]. Liposomal formulations have been studied for the treatment of *S. pneumonia* biofilms in vitro. Silva, et al. [153] used deformable liposomes to deliver and improve the bioavailability of the anti-bacterial/-biofilm endolysin MSlys for in vitro *S. pneumonia* treatment Endolysins, hydrolases produced by bacteriophages to disrupt the bacterial peptidoglycan cell wall, are being studied to replace or assist antibiotics [153,154]. The liposomes had relatively high encapsulation efficiency and loading capacity and a sustained release without any initial burst release. The deformable liposomal MSlys reduced biofilms comparably to free MSlys and significantly higher than less deformable liposomes loaded with MSlys [153].

Liposomal formulations have also been studied with the *Streptococcus oralis* (*S. oralis*) and *mutans* (*S. mutans*) biofilm-forming strains. *S. oralis* can form biofilms in critical locations such as implants [155], while *S. mutans* develop biofilms at sites such as tooth surfaces in which they form dental plaques [156]. Harper, et al. [45] reported the use of positively and negatively charged electrolytes with antibacterial anionic α-TP and assessed their ability to diffuse through biofilms of *S. oralis* and *S. mutans*. Unlike the negatively charged phosphate buffer solution, which failed to penetrate the biofilms, the positively charged TRIS buffer diffused through the *S. oralis* and *S. mutans* biofilms and inhibited their growth. The observed infectivity of the negative buffer was due to repulsion with the negatively charged biofilm surface, unlike the case with the cationic electrolyte. However, the anti-biofilm effect was less pronounced against *S. mutans* than *S. oralis* [45].

### 6.5. Other Gram-Positive Species Biofilms

Biofilms produced by *C. acnes* were explored for treatment via antimicrobial enzymes delivered with SME-incorporated cationic liposomal vehicles. DNase I and proteinase K, enzymes that prevent biofilm formation and disrupt formed biofilms, delivered using cationic liposomes, prevented the growth of *C. acnes* biofilms to a degree surpassing that of the free enzymes and single enzyme-loaded liposomes. Furthermore, the liposomal composition diffused through the majority (~85%) of the formed biofilm. Notably, the enzyme-loaded liposomes maintained their efficacy in vivo, eradicating *C. acnes* grown on catheters [157].

Other gram-positive strains with the potential to form biofilms that have been used to assess the effectivity of anti-biofilm liposome-based formulations include *Bacillus subtilis*, *Mycobacterium avium*, *Mycobacterium avium* subsp. *hominissuis*, *Mycobacterium abscessus*, and *Listeria monocytogenes* (*L. monocytogenes*). Liposomes carrying a novel hydrophobic compound that can inhibit energy-coupling factor transporters were reported to reduce *Bacillus subtilis* biofilms. Notable elimination of bacterial cells on the surface of the biofilm was observed with the loaded and unloaded drug as well as the antibiotic gentamicin at the same concentration. However, the liposomal drug was not as effective in eliminating cells within the matrix [158]. Liposomes delivering the antibiotic ciprofloxacin significantly eradicated *Mycobacterium abscessus* and *Mycobacterium avium* subsp. *hominissuis* biofilms compared to the free drug [159]. Zhang, et al. [160] delivered the antibiotic amikacin to *Mycobacterium avium* through a liposomal inhalation formulation and achieved efficient infiltration into the *Mycobacterium avium* biofilm and dose-dependent reduction in biofilm viable cells [160].

As for *L. monocytogenes*, liposomes were studied with incorporated antimicrobial agents, including surfactants [100], peptides [161], and antibiotics [95]. The antimicrobial surfactant rhamnolipid was incorporated into liposomes encapsulating nisin, which kills bacteria by disrupting their cell wall synthesis to inhibit *L. monocytogenes* biofilm. This formulation inhibited 50% of the *L. monocytogenes* biofilm and reduced them by up to 84% in vitro [100]. Furthermore, cationic liposomes incorporating the natural antibacterial Alpep7 peptide exhibited targeting towards biofilm activity, improved adsorption onto the biofilm, and an anti-biofilm activity at a concentration lower than that of the free Alpep7. Regrowth analysis indicated higher anti-biofilm activity of the liposomal formulation compared to unencapsulated Alpep7 [161]. Phosphatidylcholine-modified chitosan/liposome nanocarriers loaded with the antibiotic gentamicin destroyed and eliminated *L. monocytogenes* biofilm via antibiotic penetration into the biofilm [95].

**Table 3:** Summary of liposomal drug delivery systems against gram-positive bacteria (not active (-); active (+); very active (++)).

Bacterial Strain	Liposome Type	Active Compound	Dosage	Efficiency	Refs.
MRSA	Glycosylated cationic liposomes	Trans-resveratrol	1.2 mM MIC	Galactosylated liposomes (++) Mannosylated liposomes (+) Glucosylated liposomes (-)	[35]
	Mannose-modified liposomes	Platensimycin	0.5 to 8 μg/mL	Mannose-modified liposomal platensimycin and control (+)	[126]
	Liposome microbubbles	HBD-3	-	Liposomal formulations (+)	[72]
	Neutral Liposomes	SME	0.1 mg/mL SME	Liposomal formulations (+)	[32]
	Multilamellar liposomes	Lipopeptide surfactant + copper	105 μg/mL	Liposomal formulations (+)	[44]
	Neutral liposomes	Biosurfactant	1.25–5 mg/mL biosurfactant	Liposomal formulations (+)	[131]
	Conventional neutral, deformable, propylene glycol, and cationic liposomes	Azithromycin	0.5–8 µg/mL MBIC	Cationic liposomes (++) neutral, deformable, and propylene glycol liposomes (+)	[34]
	Neutral liposomes	Berberine + curcumin	8 µg/mL berberine + 10 µg/mL curcumin MIC	Liposomal formulations (+)	[128]
	Stimuli-responsive multilamellar liposomes	Vancomycin	0.97 μg/mL	Liposomal formulations (+)	[132]
MSSA and MRSA	Conventional neutral, fusogenic, and cationic LUVs	Vancomycin	0.78–1.56 µg/mL MIC	Fusogenic liposomes (++) Cationic and neutral liposomes (+)	[39]
MSSA	Stealth liposomes	Nafcillin	0.5 μg/mL PEGylated formulation MBIC 1 μg/mL unPEGylated formulation MBIC	PEGylated liposomes (++) UnPEGylated liposomes (+)	[133]
MSSA ATCC	Cationic, anionic, and neutral liposomes	Rifabutin	0.006 µg/mL MIC	Liposomal formulations (+)	[134]
Vancomycin-intermediate *S. aureus*	Stimuli-responsive liposome	Tungsten sulfide quantum dots + vancomycin	50 μg/mL	Liposomal formulations (+)	[68]
*Staphylococcus aureus*	Immunoliposomes	Isosorbide mononitrate and anti-α-hemolysin	45 mg/mL	Isosorbide mononitrate immunoliposomes (++) Control (+)	[59]
	Neutral liposomes	Commercial ozonated sunflower oil “Ozodrop” and “Ozodrop gel”	20% Ozodrop 20% Ozodrop gel	Liposomal formulations (+)	[109]
	Chitosan/gum arabic-coated liposomes	Alizarin	2, 10, 20, and 50 μg/mL	Liposomal formulations (+)	[143]
	Neutral Liposomes	Copper	Unmentioned	Liposomal formulations (-)	[96]
	Neutral liposomes	Toluidine Blue O	5, 10, and 25 ppm	Liposomal formulations (+)	[142]
	Cationic liposomes	Cefquinome sulfate	0.48 μg/mL MIC, and 0.5, 1.5, 2, and 2.5 of the MIC	Liposomal formulations (+)	[136]
	Neutral liposomes	Levofloxacin and serratiopeptidase	1/16, 1/8, ¼, and ½ of 4 μg/mL levofloxacin (MIC)+50 μg/mL serratiopeptidase	Liposomal formulations (+)	[137]
	Gold nanoparticle-coated liposomes	Curcumin	200 µg/mL	Liposomal formulations (+)	[140]
	Anionic liposomes	Lysozyme and gentamicin (resulting in cationic formulation)	Unmentioned	Liposomal formulations (+)	[84]
	Neutral liposomes	Lysozyme and levofloxacin	Unmentioned	Liposomal formulations (-)	[139]
*Staphylococcus aureus*	Neutral liposomes	Azithromycin	4 μg/mL MIC	Liposomal formulations: against biofilm formation (+) against preformed biofilm (-)	[90]
*Staphylococcus aureus*	Phospholipid-porphyrin liposome-like assemblies	Photosensitizer	100 μM photosensitizer	Liposomal formulations (+)	[67]
*Staphylococcus aureus*	Neutral liposomes coated with chitosan hydrogel	Chlorhexidine	Unmentioned	Liposomal formulations (+)	[94]
*Staphylococcus aureus*	Stimuli-responsive liposomes	LysRODI	40 µg/mL LysRODI	Liposomal formulations (+)	[141]
*Staphylococcus aureus*	Stimuli-responsive liposomes	Vancomycin, rifampicin, and SA31 aptamer	25 μg/mL MBEC	Liposomal formulations (+)	[60]
*Streptococcus epidermidis*	Liposomes incorporated into hydrogels	Tetracycline HCl and tretinoin	0.25 µg/mL MBIC	Liposomal formulations (+)	[145]
	Cationic liposomes	Rifampin and N-acetylcysteine	Unmentioned	Liposomal rifampin/N-acetylcysteine or N-acetylcysteine: against formed formation (+) against biofilm formation (-) Control: against formed biofilm and biofilm formation (+)	[144]
	Neutral liposomes	Moxifloxacin	43 µM lipid + 0.3 µM moxifloxacin	Liposomal formulations (+)	[146]
	Neutral liposomes	Vancomycin and C2DA	0.83 mg/mL vancomycin + 0.64 mg/mL C2DA	Liposomal formulations (+)	[147]
*Staphylococcus epidermidis*	Glycosylated cationic liposomes	Usnic acid	1 mM total lipids + 0.05 mM (MIC) or 0.01 mM (1/5 MIC) usnic acid	Liposomal formulations (+)	[148]
*Staphylococcus saprophyticus* subspecies *bovis*	Neutral liposomes	Hypericin and (Hyp-HPβCD)	Unmentioned	Liposomal formulations (+)	[149]
	Neutral liposomes	Hypericin and HPβCD	40 µM	Liposomal formulations (+)	[150]
*S. pneumonaie*	Deformable stealth or sodium cholate liposomes	Endolysin MSlys	4 μM MSlys	Stealth liposomes (++) Sodium cholate liposomes (+)	[153]
*S. oralis* and *S. mutans*	Anionic liposomes	α-TP	(+) α-TP 0.8 mM	In positive buffer (+) In negative buffer (-)	[45]
*C. acnes*	Cationic liposomes	SME, DNase I, and proteinase K	150 μg/mL SME + 25 μg/mL DNase I + 10 μg/mL proteinase K	Liposomal formulations (+)	[157]
*Bacillus subtilis*	Neutral liposomes	Energy-coupling factor transporter inhibitor (HIPS5031)	9.64 µM	Liposomal formulations (+)	[158]
*Mycobacterium avium*	Neutral liposomes	Amikacin	>16 μg/mL	Liposomal formulations (+)	[160]
*Mycobacterium avium* subsp. *hominissuis* and *Mycobacterium abscessus*	Neutral liposomes	Ciprofloxacin	50 and 100 μg/mL	Liposomal formulations (+)	[159]
*Listeria monocytogenes* and *Staphylococcus aureus*	Neutral liposomes	Rhamnolipid and nisin	*Listeria monocytogenes*: 2.5 μg/mL MIC *Staphylococcus aureus*: 1.25 μg/mL MIC	Liposomal formulations (+)	[100]
*Listeria monocytogenes*	Cationic liposomes	Alpep7 protein	52.78 μg/mL (4 μg/mL of Alpep7)	Liposomal formulations (+)	[161]
*Listeria monocytogenes*	Liposomal chitosan	Gentamicin	20 μg/mL gentamicin	Liposomal formulations (+)	[95]

## 7. Literature Gaps in the Treatment of Gram-Negative and Gram-Positive Biofilms Using Liposomal Systems

The available recent literature provides promising results on the future of the use of liposome-based delivery systems for the treatment of gram-negative and gram-positive biofilms. This is especially true for *P. aeruginosa*, *E. coli*, and *S. aureus* strains, on which most of the literature is currently available. However, other medically salient strains should be incorporated in studies investigating liposomal formulations against biofilms such as *Mycobacterium tuberculosis* and *Neisseria gonorrhoeae.* Available data is still lacking in some areas and requires to be supplemented by certain information. For instance, most of the studies discussed here assessed the anti-biofilm activity of the liposomal formulations in vitro only without in vivo data. Furthermore, it should be pinpointed whether the observed antibiofilm activity of these formulations would be retained against real-time performed environmental biofilms where many other factors are involved, namely humidity, temperature, nutrient availability, pH, presence of detergents, and even interactions with other microorganisms [162]. This would provide a more tangible assessment of their efficacy, as real-time situations are rarely this simple.

Additionally, the studies discussed here investigate biofilms of single strains. However, no studies evaluate the activity of the liposomal formulations against multi-strain biofilms that are composed of multiple different bacterial strains, which most environmental biofilms are [163,164]. Even fewer studies investigated the effects of liposomal formulations against biofilm-mediated clinical cases and, if available, in murine models. Furthermore, although several studies assess the biocompatibility of the liposomal systems, only a few assess its hemocompatibility and systematic toxicity and the involvement of the immune system, which are necessary parameters to accurately assess the feasibility of these formulations for biofilm treatment in clinical cases.

Another important point that has been overlooked is the effect the bacterial gram stain has on the efficacy of the liposomal formulation. Studies merely mentioned the interactions between the liposomal formulations and the negatively charged bacterial biofilm, paying no regard to the gram stain of the studied strain [34,44]. This is of importance, especially given the superior resistance of gram-negative bacteria [75]. Future studies should investigate the interactions between the LPS and the thinner peptidoglycan layer characterizing gram-negative bacteria and compare them with those of gram-positive strains [76].

Finally, despite the extensive exploration of stimuli-responsive liposomes for other biomedical purposes, such as cancer theragnostic [165], conventional liposomal systems seem to be the main archetype studied for gram-positive and gram-negative biofilm treatment. Furthermore, only a few nanoparticles have been studied with liposomes for biofilm treatment via multimodal modal approaches. Other nanoparticles that could potentially be explored include, for example, iron oxide, capable of photothermal therapy [166] and silver nanoparticles, capable of both photothermal and photodynamic therapy [167,168]. Such nanoparticles can be used for the hyperthermia-induced killing of the biofilm cells and heat-triggered drug release from liposomes.

## 8. Conclusions

Residing within biofilms allows bacteria to survive in conditions in which they would otherwise wither. Moreover, biofilm-bound bacteria are up to 1000-fold more resistant to antibiotic agents [2,3]. Thus, targeting bacterial biofilm formation is deemed a priority. However, biofilms are not an easy target. With their amphiphilic nature, liposomes can bear various hydrophilic and hydrophobic compounds. This makes liposomes excellent candidates for drug delivery, specifically to target biofilm-bound bacteria. Accordingly, we envisioned outlining the efficacy of liposomal formulations against biofilms of pathogenic gram-negative and gram-positive bacteria.

In our review, we came across a variety of liposomal formulations with promising antibiofilm efficiency against pathogenic gram-negative strains. Against *E. coli* and *P. aeruginosa*, stimuli-responsive, conventional (neutral and charged liposomes) displayed antibiofilm potency and enhanced that of encapsulated antibiotics. As for Stimuli-responsive liposomes’ ability to enhance antibiotics’ antibiofilm activity against *P. aeruginosa*, there remains a lack of consensus. Aside from *P. aeruginosa*, chitosan formulations also proved efficacious against *A. baumannii*, *Porphyromonas gingivalis*, and *Prevotella intermedia* biofilms. Interestingly, *Salmonella* species have been targeted by compounds isolated from essential oils such as geraniol, thymol, and carvacrol; their biofilms proving susceptible. Biofilms of the *Klebsiella* genus were also reported to be susceptible to conventional and charged antibiotic-bearing liposomes. Liposomal curcumin was observed to be active against *Aeromonas hydrophila*, *Aeromonas sobria*, and *Serratia grimesii*.

Similarly, various formulations exhibited antibiofilm activity against pathogenic gram-positive strains. A repertoire of conventional (neutral or charged) and stimuli liposomes devoid and bearing antibiotics proved efficacious against staphylococcus aureus, including MRSA, MSSA, and Vancomycin-intermediate *S. aureus*. As for *S. epidermis*, glycosylated, cationic, hydrogel-incorporated, and antibiotic liposomal formulations were observed to target its biofilms. Other Staphylococci, namely *S. saprophyticus* subspecies bovis, *S. pneumonaie*, *S. oralis*, and *S. mutans* proved susceptible to liposomal administration. Yet so were *C. acnes* and *Bacillus subtilis.* Notably, *Mycobacterium avium* and *Mycobacterium abscessus* biofilms were impacted by liposomal formulations of antibiotics and antibacterial peptides such as nisin and ciprofloxacin. *Listeria monocytogenes* biofilms were successfully targeted by cationic, chitosan, and nisin-bearing liposomes.

Overall, this review highlights the potential of liposomal formulations against pathogenic bacterial biofilms, propelling their incorporation in further studies in the quest to find novel antibiofilm measures. However, other medically salient strains should be targets of further investigations, specifically strains with well-developed resistance, such as *Mycobacterium tuberculosis* and *Neisseria gonorrhoeae.* Since studies overlooked the effect of the gram-stain of bacterial strains on liposomal efficiency, mechanistic and comparative studies should be conducted to create liposomal formulations catering for the compositional differences of both stains, especially the more resistant, the gram-negative. Most importantly, with such an escalating emergence rate of antibiotic resistance, most bacterial species are worthy of incorporation in studies, specifically environmental species which, upon acquiring resistance, we will fail to evade.

## Figures and Tables

**Table 1:** Types and advantages of liposomes for drug delivery purposes.

Liposome Type	Description	Advantages	Limitations	Refs.
Conventional (cationic, anionic, neutral)	Bilayer composed of neutral, positively or negatively charged phospholipids	Reduced drug-associated toxicity	Rapid removal from circulation	[41,42]
Deformable (elastic)	Elastic lipid bilayer	Deeper skin penetration for topical applications	Cargo release during prolonged storage times	[47]
Stealth	PEG-coated liposomes	Prolonged circulation time	Non-biodegradability of high molar mass PEG and toxicity of low molar mass PEG	[42,48]
Targeted	Liposomes surface modified with targeting moieties such as antibodies	Specificity	Rapid elimination from the bloodstream	[54]
Stimuli-responsive	Liposomes change conformation and release encapsulated cargo in response to external and internal triggers	Reduced off-target toxicity	*	[43,63,64]

* varies depending on stimuli used.

## Data Availability

Not applicable.

## Abbreviations

6-AIH	2-aminomidazole derivative
6-NIH	6-(2-nitroimidazole) hexylamine
α-TP	alpha-tocopherol phosphate
BC	Betainylated Cholesterol
C2DA	cis-2-decenoic acid
DODAB	dimethyldioctadecylammonium bromide
DOPE	1,2-dioleoyl-sn-glycero-3-phosphoethanolamine
DOTAC	dimethyldioctadecyl-ammonium chloride
DOTAP	2,3-dioleoyloxy-propyl)-trimethylammonium-chloride
DPPC	dipalmitoylphosphatidyl-choline cholesterol
DSPC	distearoylphosphtidylcholine
DVDMS	nano-Sinoporphyrin sodium
DVDMS	Sinoporphyrin sodium
HBD-3	Human beta-Defensin-3
Hyp-HPβCD	(2-hydroxypropyl)-β-cyclodextrine
LysRODI	antistaphylococcal endolysin
MTAB	(11-mercaptoundecyl)-N,N,N-trimethylammonium bromide
PAβN	phenylalanine arginine beta-naphthylamide
PGP-me	2,3-di-O-phytanyl-sn-glycero-1-phospho-(3′-sn-glycerol-1′-methylphosphate)
SME	soyaethyl morpholinium ethosulfate
TMC	N,N,N-trimethyl chitosan
TRIS	tris-(hydroxymethyl)aminomethane

## References

[1] Bai X., Nakatsu C.H., Bhunia A.K. (2021). Bacterial Biofilms and Their Implications in Pathogenesis and Food Safety. Foods.

[2] Srinivasan R., Santhakumari S., Poonguzhali P., Geetha M., Dyavaiah M., Xiangmin L. (2021). Bacterial Biofilm Inhibition: A Focused Review on Recent Therapeutic Strategies for Combating the Biofilm Mediated Infections. Front. Microbiol..

[3] Ramadan R., Omar N., Dawaba M., Moemen D. (2021). Bacterial Biofilm Dependent Catheter Associated Urinary Tract Infections: Characterization, Antibiotic Resistance Pattern and Risk Factors. Egypt. J. Basic Appl. Sci..

[4] Bi Y., Xia G., Shi C., Wan J., Liu L., Chen Y., Wu Y., Zhang W., Zhou M., He H. (2021). Therapeutic Strategies against Bacterial Biofilms. Fundam. Res..

[5] An A.Y., Choi K.-Y.G., Baghela A.S., Hancock R.E.W. (2021). An Overview of Biological and Computational Methods for Designing Mechanism-Informed Anti-Biofilm Agents. Front. Microbiol..

[6] Singh A., Amod A., Pandey P., Bose P., Pingali M.S., Shivalkar S., Varadwaj P.K., Sahoo A.K., Samanta S.K. (2022). Bacterial Biofilm Infections, Their Resistance to Antibiotics Therapy and Current Treatment Strategies. Biomed. Mater..

[7] Melander R.J., Basak A.K., Melander C. (2020). Natural Products as Inspiration for the Development of Bacterial Antibiofilm Agents. Nat. Prod. Rep..

[8] Wang D.-Y., van der Mei H.C., Ren Y., Busscher H.J., Shi L. (2020). Lipid-Based Antimicrobial Delivery-Systems for the Treatment of Bacterial Infections. Front. Chem..

[9] Larsson D.G.J., Flach C.-F. (2022). Antibiotic Resistance in the Environment. Nat. Rev. Microbiol..

[10] Anu Mary Ealia S., Saravanakumar M.P. (2017). A Review on the Classification, Characterisation, Synthesis of Nanoparticles and Their Application. IOP Conf. Ser. Mater. Sci. Eng..

[11] Ijaz I., Gilani E., Nazir A., Bukhari A. (2020). Detail Review on Chemical, Physical and Green Synthesis, Classification, Characterizations and Applications of Nanoparticles. Green Chem. Lett. Rev..

[12] Khan I., Saeed K., Khan I. (2019). Nanoparticles: Properties, Applications and Toxicities. Arab. J. Chem..

[13] Ferreira M., Ogren M., Dias J.N.R., Silva M., Gil S., Tavares L., Aires-da-Silva F., Gaspar M.M., Aguiar S.I. (2021). Liposomes as Antibiotic Delivery Systems: A Promising Nanotechnological Strategy against Antimicrobial Resistance. Molecules.

[14] Rukavina Z., Vanić Ž. (2016). Current Trends in Development of Liposomes for Targeting Bacterial Biofilms. Pharmaceutics.

[15] Wang Y. (2021). Liposome as a Delivery System for the Treatment of Biofilm-mediated Infections. J. Appl. Microbiol..

[16] Hu D., Zou L., Yu W., Jia F., Han H., Yao K., Jin Q., Ji J. (2020). Relief of Biofilm Hypoxia Using an Oxygen Nanocarrier: A New Paradigm for Enhanced Antibiotic Therapy. Adv. Sci..

[17] Yang L., Liu Y., Wu H., Høiby N., Molin S., Song Z. (2011). Current Understanding of Multi-species Biofilms. Int. J. Oral Sci..

[18] Nadar S., Khan T., Patching S.G., Omri A. (2022). Development of Antibiofilm Therapeutics Strategies to Overcome Antimicrobial Drug Resistance. Microorganisms.

[19] Abebe G.M. (2020). The Role of Bacterial Biofilm in Antibiotic Resistance and Food Contamination. Int. J. Microbiol..

[20] Vestby L.K., Grønseth T., Simm R., Nesse L.L. (2020). Bacterial Biofilm and Its Role in the Pathogenesis of Disease. Antibiotics.

[21] Gebreyohannes G., Nyerere A., Bii C., Sbhatu D.B. (2019). Challenges of Intervention, Treatment, and Antibiotic Resistance of Biofilm-Forming Microorganisms. Heliyon.

[22] Jamal M., Ahmad W., Andleeb S., Jalil F., Imran M., Nawaz M.A., Hussain T., Ali M., Rafiq M., Kamil M.A. (2018). Bacterial Biofilm and Associated Infections. J. Chin. Med. Assoc..

[23] Mahamuni-Badiger P.P., Patil P.M., Badiger M.V., Patel P.R., Thorat-Gadgil B.S., Pandit A., Bohara R.A. (2020). Biofilm Formation to Inhibition: Role of Zinc Oxide-Based Nanoparticles. Mater. Sci. Eng. C.

[24] Hawas S., Verderosa A.D., Totsika M. (2022). Combination Therapies for Biofilm Inhibition and Eradication: A Comparative Review of Laboratory and Preclinical Studies. Front. Cell. Infect. Microbiol..

[25] Ghosh A., Jayaraman N., Chatterji D. (2020). Small-Molecule Inhibition of Bacterial Biofilm. ACS Omega.

[26] Verderosa A.D., Totsika M., Fairfull-Smith K.E. (2019). Bacterial Biofilm Eradication Agents: A Current Review. Front. Chem..

[27] Haney E.F., Trimble M.J., Hancock R.E.W. (2021). Microtiter Plate Assays to Assess Antibiofilm Activity against Bacteria. Nat. Protoc..

[28] Fu J., Zhang Y., Lin S., Zhang W., Shu G., Lin J., Li H., Xu F., Tang H., Peng G. (2021). Strategies for Interfering with Bacterial Early Stage Biofilms. Front. Microbiol..

[29] Khan F., Pham D.T.N., Oloketuyi S.F., Manivasagan P., Oh J., Kim Y.-M. (2020). Chitosan and Their Derivatives: Antibiofilm Drugs against Pathogenic Bacteria. Colloids Surf. B Biointerfaces.

[30] Lin Y.-K., Yang S.-C., Hsu C.-Y., Sung J.-T., Fang J.-Y. (2021). The Antibiofilm Nanosystems for Improved Infection Inhibition of Microbes in Skin. Molecules.

[31] Dos Santos Ramos M.A., Da Silva P., Spósito L., De Toledo L., Bonifácio B., Rodero C.F., Dos Santos K., Chorilli M., Bauab T.M. (2018). Nanotechnology-Based Drug Delivery Systems for Control of Microbial Biofilms: A Review. Int. J. Nanomed..

[32] Lin M.-H., Hung C.-F., Aljuffali I.A., Sung C.T., Huang C.-T., Fang J.-Y. (2017). Cationic Amphiphile in Phospholipid Bilayer or Oil–Water Interface of Nanocarriers Affects Planktonic and Biofilm Bacteria Killing. Nanomed. Nanotechnol. Biol. Med..

[33] Nsairat H., Khater D., Sayed U., Odeh F., Al Bawab A., Alshaer W. (2022). Liposomes: Structure, Composition, Types, and Clinical Applications. Heliyon.

[34] Rukavina Z., Šegvić Klarić M., Filipović-Grčić J., Lovrić J., Vanić Ž. (2018). Azithromycin-Loaded Liposomes for Enhanced Topical Treatment of Methicillin-Resistant *Staphyloccocus Aureus* (MRSA) Infections. Int. J. Pharm..

[35] Aiello S., Pagano L., Ceccacci F., Simonis B., Sennato S., Bugli F., Martini C., Torelli R., Sanguinetti M., Ciogli A. (2021). Mannosyl, Glucosyl or Galactosyl Liposomes to Improve Resveratrol Efficacy against Methicillin Resistant *Staphylococcus Aureus* Biofilm. Colloids Surf. A Physicochem. Eng. Asp..

[36] Alzahrani N.M., Booq R.Y., Aldossary A.M., Bakr A.A., Almughem F.A., Alfahad A.J., Alsharif W.K., Jarallah S.J., Alharbi W.S., Alsudir S.A. (2022). Liposome-Encapsulated Tobramycin and IDR-1018 Peptide Mediated Biofilm Disruption and Enhanced Antimicrobial Activity against *Pseudomonas Aeruginosa*. Pharmaceutics.

[37] Kluzek M., Oppenheimer-Shaanan Y., Dadosh T., Morandi M.I., Avinoam O., Raanan C., Goldsmith M., Goldberg R., Klein J. (2022). Designer Liposomic Nanocarriers Are Effective Biofilm Eradicators. ACS Nano.

[38] Nicolosi D., Scalia M., Nicolosi V.M., Pignatello R. (2010). Encapsulation in Fusogenic Liposomes Broadens the Spectrum of Action of Vancomycin against Gram-Negative Bacteria. Int. J. Antimicrob. Agents.

[39] Scriboni A.B., Couto V.M., de Morais Ribeiro L.N., Freires I.A., Groppo F.C., de Paula E., Franz-Montan M., Cogo-Müller K. (2019). Fusogenic Liposomes Increase the Antimicrobial Activity of Vancomycin Against *Staphylococcus Aureus* Biofilm. Front. Pharmacol..

[40] Zahra M.-J., Hamed H., Mohammad R.-Y., Nosratollah Z., Akbarzadeh A., Morteza M. (2017). Evaluation and Study of Antimicrobial Activity of Nanoliposomal Meropenem against *Pseudomonas Aeruginosa* Isolates. Artif. Cells Nanomed. Biotechnol..

[41] Sercombe L., Veerati T., Moheimani F., Wu S.Y., Sood A.K., Hua S. (2015). Advances and Challenges of Liposome Assisted Drug Delivery. Front. Pharmacol..

[42] Vassallo A., Silletti M.F., Faraone I., Milella L. (2020). Nanoparticulate Antibiotic Systems as Antibacterial Agents and Antibiotic Delivery Platforms to Fight Infections. J. Nanomater..

[43] Antoniou A.I., Giofrè S., Seneci P., Passarella D., Pellegrino S. (2021). Stimulus-Responsive Liposomes for Biomedical Applications. Drug Discov. Today.

[44] Kannan S., Solomon A., Krishnamoorthy G., Marudhamuthu M. (2021). Liposome Encapsulated Surfactant Abetted Copper Nanoparticles Alleviates Biofilm Mediated Virulence in Pathogenic *Pseudomonas Aeruginosa* and MRSA. Sci. Rep..

[45] Harper R.A., Carpenter G.H., Proctor G.B., Harvey R.D., Gambogi R.J., Geonnotti A.R., Hider R., Jones S.A. (2019). Diminishing Biofilm Resistance to Antimicrobial Nanomaterials through Electrolyte Screening of Electrostatic Interactions. Colloids Surf. B Biointerfaces.

[46] Elsayed M.M.A., Abdallah O.Y., Naggar V.F., Khalafallah N.M. (2006). Deformable Liposomes and Ethosomes: Mechanism of Enhanced Skin Delivery. Int. J. Pharm..

[47] Nayak D., Tippavajhala V.K. (2021). A Comprehensive Review on Preparation, Evaluation and Applications of Deformable Liposomes. Iran. J. Pharm. Res..

[48] Knop K., Hoogenboom R., Fischer D., Schubert U.S. (2010). Poly(Ethylene Glycol) in Drug Delivery: Pros and Cons as Well as Potential Alternatives. Angew. Chem. Int. Ed..

[49] Gabizon A., Catane R., Uziely B., Kaufman B., Safra T., Cohen R., Martin F., Huang A., Barenholz Y. (1994). Prolonged Circulation Time and Enhanced Accumulation in Malignant Exudates of Doxorubicin Encapsulated in Polyethylene-Glycol Coated Liposomes. Cancer Res..

[50] Ghaferi M., Raza A., Koohi M., Zahra W., Akbarzadeh A., Ebrahimi Shahmabadi H., Alavi S.E. (2022). Impact of PEGylated Liposomal Doxorubicin and Carboplatin Combination on Glioblastoma. Pharmaceutics.

[51] Maruyama K., Yuda T., Okamoto A., Kojima S., Suginaka A., Iwatsuru M. (1992). Prolonged Circulation Time in Vivo of Large Unilamellar Liposomes Composed of Distearoyl Phosphatidylcholine and Cholesterol Containing Amphipathic Poly(Ethylene Glycol). Biochim. Biophys. Acta (BBA) Lipids Lipid Metab..

[52] McNeeley K.M., Annapragada A., Bellamkonda R.V. (2007). Decreased Circulation Time Offsets Increased Efficacy of PEGylated Nanocarriers Targeting Folate Receptors of Glioma. Nanotechnology.

[53] Zhang L., Han L., Sun X., Gao D., Qin J., Wang J. (2012). The Use of PEGylated Liposomes to Prolong the Circulation Lifetime of Salvianolic Acid B. Fitoterapia.

[54] Bendas G. (2001). Immunoliposomes: A Promising Approach to Targeting Cancer Therapy. BioDrugs.

[55] Gholizadeh S., Dolman E.M., Wieriks R., Sparidans R.W., Hennink W.E., Kok R.J. (2018). Anti-GD2 Immunoliposomes for Targeted Delivery of the Survivin Inhibitor Sepantronium Bromide (YM155) to Neuroblastoma Tumor Cells. Pharm. Res..

[56] Huwyler J., Wu D., Pardridge W.M. (1996). Brain Drug Delivery of Small Molecules Using Immunoliposomes. Proc. Natl. Acad. Sci. USA.

[57] Loureiro J.A., Gomes B., Fricker G., Cardoso I., Ribeiro C.A., Gaiteiro C., Coelho M.A.N., do Carmo Pereira M., Rocha S. (2015). Dual Ligand Immunoliposomes for Drug Delivery to the Brain. Colloids Surf. B Biointerfaces.

[58] Robinson A.M., Creeth J.E., Jones M.N. (2000). The Use of Immunoliposomes for Specific Delivery of Antimicrobial Agents to Oral Bacteria Immobilized on Polystyrene. J. Biomater. Sci. Polym. Ed..

[59] Zhang Y., Zhao Y., Dong D., Li X., Li Z., Li S., Wang J. (2019). Effects of Isosorbide Mononitrate Loaded Nanoparticles Conjugated with Anti-Staphylococcus Aureus A-toxin on Staphylococcus Aureus Biofilms. Exp. Med..

[60] Ommen P., Hansen L., Hansen B.K., Vu-Quang H., Kjems J., Meyer R.L. (2022). Aptamer-Targeted Drug Delivery for Staphylococcus Aureus Biofilm. Front. Cell. Infect. Microbiol..

[61] Wei Z., Zhou Y., Wang R., Wang J., Chen Z. (2022). Aptamers as Smart Ligands for Targeted Drug Delivery in Cancer Therapy. Pharmaceutics.

[62] Yang K., Gitter B., Rüger R., Wieland G.D., Chen M., Liu X., Albrecht V., Fahr A. (2011). Antimicrobial Peptide-Modified Liposomes for Bacteria Targeted Delivery of Temoporfin in Photodynamic Antimicrobial Chemotherapy. Photochem. Photobiol. Sci..

[63] Ali A.A., Al-Othman A., Al-Sayah M.H. (2022). Multifunctional Stimuli-Responsive Hybrid Nanogels for Cancer Therapy: Current Status and Challenges. J. Control. Release.

[64] Zhao X., Bai J., Yang W. (2021). Stimuli-Responsive Nanocarriers for Therapeutic Applications in Cancer. Cancer Biol. Med..

[65] Ding M., Zhao W., Song L.-J., Luan S.-F. (2022). Stimuli-Responsive Nanocarriers for Bacterial Biofilm Treatment. Rare Met..

[66] Koga K., Tagami T., Ozeki T. (2021). Gold Nanoparticle-Coated Thermosensitive Liposomes for the Triggered Release of Doxorubicin, and Photothermal Therapy Using a near-Infrared Laser. Colloids Surf. A Physicochem. Eng. Asp..

[67] Cressey P., Bronstein L.-G., Benmahmoudi R., Rosilio V., Regeard C., Makky A. (2022). Novel Liposome-like Assemblies Composed of Phospholipid-Porphyrin Conjugates with Photothermal and Photodynamic Activities against Bacterial Biofilms. Int. J. Pharm..

[68] Xu M., Hu Y., Xiao Y., Zhang Y., Sun K., Wu T., Lv N., Wang W., Ding W., Li F. (2020). Near-Infrared-Controlled Nanoplatform Exploiting Photothermal Promotion of Peroxidase-like and OXD-like Activities for Potent Antibacterial and Anti-Biofilm Therapies. ACS Appl. Mater. Interfaces.

[69] Zou L., Hu D., Wang F., Jin Q., Ji J. (2022). The Relief of Hypoxic Microenvironment Using an O2 Self-Sufficient Fluorinated Nanoplatform for Enhanced Photodynamic Eradication of Bacterial Biofilms. Nano Res..

[70] Alamoudi K., Martins P., Croissant J.G., Patil S., Omar H., Khashab N.M. (2017). Thermoresponsive Pegylated Bubble Liposome Nanovectors for Efficient SiRNA Delivery via Endosomal Escape. Nanomedicine.

[71] Fu Y.-Y., Zhang L., Yang Y., Liu C., He Y.-N., Li P., Yu X. (2019). Synergistic Antibacterial Effect of Ultrasound Microbubbles Combined with Chitosan-Modified Polymyxin B-Loaded Liposomes on Biofilm-Producing *Acinetobacter Baumannii*. Int. J. Nanomed..

[72] Zhou H., Fang S., Kong R., Zhang W., Wu K., Xia R., Shang X., Zhu C. (2018). Effect of Low Frequency Ultrasound plus Fluorescent Composite Carrier in the Diagnosis and Treatment of Methicillin-Resistant *Staphylococcus Aureus* Biofilm Infection of Bone Joint Implant. Int. J. Clin. Exp. Med..

[73] Raza A., Hayat U., Rasheed T., Bilal M., Iqbal H.M.N. (2019). “Smart” Materials-Based near-Infrared Light-Responsive Drug Delivery Systems for Cancer Treatment: A Review. J. Mater. Res. Technol..

[74] Andra V.V.S.N.L., Pammi S.V.N., Bhatraju L.V.K.P., Ruddaraju L.K. (2022). A Comprehensive Review on Novel Liposomal Methodologies, Commercial Formulations, Clinical Trials and Patents. Bionanoscience.

[75] Breijyeh Z., Jubeh B., Karaman R. (2020). Resistance of Gram-Negative Bacteria to Current Antibacterial Agents and Approaches to Resolve It. Molecules.

[76] Zhang G., Meredith T.C., Kahne D. (2013). On the Essentiality of Lipopolysaccharide to Gram-Negative Bacteria. Curr. Opin. Microbiol..

[77] Bertani B., Ruiz N. (2018). Function and Biogenesis of Lipopolysaccharides. EcoSal Plus.

[78] Alhariri M., Majrashi M.A., Bahkali A.H., Almajed F.S., Azghani A.O., Khiyami M., Alyamani E.J., Aljohani S.M., Halwani M.A. (2017). Efficacy of Neutral and Negatively Charged Liposome-Loaded Gentamicin on Planktonic Bacteria and Biofilm Communities. Int. J. Nanomed..

[79] Mena K.D., Gerba C.P., Whitacre D.M. (2009). Risk Assessment of *Pseudomonas Aeruginosa* in Water. Reviews of Environmental Contamination and Toxicology.

[80] Pang Z., Raudonis R., Glick B.R., Lin T.-J., Cheng Z. (2019). Antibiotic Resistance in *Pseudomonas Aeruginosa*: Mechanisms and Alternative Therapeutic Strategies. Biotechnol. Adv..

[81] Ibaraki H., Kanazawa T., Chien W.-Y., Nakaminami H., Aoki M., Ozawa K., Kaneko H., Takashima Y., Noguchi N., Seta Y. (2020). The Effects of Surface Properties of Liposomes on Their Activity against *Pseudomonas Aeruginosa* PAO-1 Biofilm. J. Drug Deliv. Sci. Technol..

[82] Thi M.T.T., Wibowo D., Rehm B.H.A. (2020). *Pseudomonas Aeruginosa* Biofilms. Int. J. Mol. Sci..

[83] Ghodake V., Vishwakarma J., Vavilala S.L., Patravale V. (2020). Cefoperazone Sodium Liposomal Formulation to Mitigate *P. Aeruginosa* Biofilm in Cystic Fibrosis Infection: A QbD Approach. Int. J. Pharm..

[84] Hou Y., Wang Z., Zhang P., Bai H., Sun Y., Duan J., Mu H. (2017). Lysozyme Associated Liposomal Gentamicin Inhibits Bacterial Biofilm. Int. J. Mol. Sci..

[85] Zhao Y., Dai X., Wei X., Yu Y., Chen X., Zhang X., Li C. (2018). Near-Infrared Light-Activated Thermosensitive Liposomes as Efficient Agents for Photothermal and Antibiotic Synergistic Therapy of Bacterial Biofilm. ACS Appl. Mater. Interfaces.

[86] Chatterjee S., Chi L., Hui P. (2019). Review of Stimuli-Responsive Polymers in Drug Delivery and Textile Application. Molecules.

[87] Mai B., Gao Y., Li M., Jia M., Liu S., Wang X., Zhang K., Liu Q., Wang P. (2021). Tailoring the Cationic Lipid Composition of Lipo-DVDMS Augments the Phototherapy Efficiency of Burn Infection. Biomater. Sci..

[88] Munaweera I., Shaikh S., Maples D., Nigatu A.S., Sethuraman S.N., Ranjan A., Greenberg D.E., Chopra R. (2018). Temperature-Sensitive Liposomal Ciprofloxacin for the Treatment of Biofilm on Infected Metal Implants Using Alternating Magnetic Fields. Int. J. Hyperth..

[89] Teirlinck E., Barras A., Liu J., Fraire J.C., Lajunen T., Xiong R., Forier K., Li C., Urtti A., Boukherroub R. (2019). Exploring Light-Sensitive Nanocarriers for Simultaneous Triggered Antibiotic Release and Disruption of Biofilms Upon Generation of Laser-Induced Vapor Nanobubbles. Pharmaceutics.

[90] Altube M.J., Martínez M.M.B., Malheiros B., Maffía P.C., Barbosa L.R.S., Morilla M.J., Romero E.L. (2020). Fast Biofilm Penetration and Anti-PAO1 Activity of Nebulized Azithromycin in Nanoarchaeosomes. Mol. Pharm..

[91] Rao Y., Sun Y., Li P., Xu M., Chen X., Wang Y., Chen Y., Deng X., Yu S., Hu H. (2022). Hypoxia-Sensitive Adjuvant Loaded Liposomes Enhance the Antimicrobial Activity of Azithromycin via Phospholipase-Triggered Releasing for Pseudomonas Aeruginosa Biofilms Eradication. Int. J. Pharm..

[92] Ben J., Zhu X., Zhang H., Chen Q. (2015). Class A1 Scavenger Receptors in Cardiovascular Diseases: SR-A1 and Cardiovascular Diseases. Br. J. Pharm..

[93] Gbian D.L., Omri A. (2021). The Impact of an Efflux Pump Inhibitor on the Activity of Free and Liposomal Antibiotics against Pseudomonas Aeruginosa. Pharmaceutics.

[94] Hemmingsen L.M., Giordani B., Pettersen A.K., Vitali B., Basnet P., Škalko-Basnet N. (2021). Liposomes-in-Chitosan Hydrogel Boosts Potential of Chlorhexidine in Biofilm Eradication in Vitro. Carbohydr. Polym..

[95] Qiu Y., Xu D., Sui G., Wang D., Wu M., Han L., Mu H., Duan J. (2020). Gentamicin Decorated Phosphatidylcholine-Chitosan Nanoparticles against Biofilms and Intracellular Bacteria. Int. J. Biol. Macromol..

[96] Sarcina L., García-Manrique P., Gutiérrez G., Ditaranto N., Cioffi N., Matos M., del Carmen Blanco-López M. (2020). Cu Nanoparticle-Loaded Nanovesicles with Antibiofilm Properties. Part I: Synthesis of New Hybrid Nanostructures. Nanomaterials.

[97] Hallan S.S., Marchetti P., Bortolotti D., Sguizzato M., Esposito E., Mariani P., Trapella C., Rizzo R., Cortesi R. (2020). Design of Nanosystems for the Delivery of Quorum Sensing Inhibitors: A Preliminary Study. Molecules.

[98] Beadell B.A., Chieng A., Parducho K.R., Dai Z., Ho S.O., Fujii G., Wang Y., Porter E. (2021). Nano- and Macroscale Imaging of Cholesterol Linoleate and Human Beta Defensin 2-Induced Changes in Pseudomonas Aeruginosa Biofilms. Antibiotics.

[99] Aljihani S.A., Alehaideb Z., Alarfaj R.E., Alghoribi M.F., Akiel M.A., Alenazi T.H., Al-Fahad A.J., Al Tamimi S.M., Albakr T.M., Alshehri A. (2020). Enhancing Azithromycin Antibacterial Activity by Encapsulation in Liposomes/Liposomal-N-Acetylcysteine Formulations against Resistant Clinical Strains of Escherichia Coli. Saudi J. Biol. Sci..

[100] Niaz T., Shabbir S., Noor T., Imran M. (2019). Antimicrobial and Antibiofilm Potential of Bacteriocin Loaded Nano-Vesicles Functionalized with Rhamnolipids against Foodborne Pathogens. LWT.

[101] Boccalini G., Conti L., Montis C., Bani D., Bencini A., Berti D., Giorgi C., Mengoni A., Valtancoli B. (2017). Methylene Blue-Containing Liposomes as New Photodynamic Anti-Bacterial Agents. J. Mater. Chem. B.

[102] Vanić Ž., Rukavina Z., Manner S., Fallarero A., Uzelac L., Kralj M., Amidžić Klarić D., Bogdanov A., Raffai T., Virok D.P. (2019). Azithromycin-Liposomes as a Novel Approach for Localized Therapy of Cervicovaginal Bacterial Infections. Int. J. Nanomed..

[103] Alarfaj R.E., Alkhulaifi M.M., Al-Fahad A.J., Aljihani S., Yassin A.E.B., Alghoribi M.F., Halwani M.A. (2022). Antibacterial Efficacy of Liposomal Formulations Containing Tobramycin and N-Acetylcysteine against Tobramycin-Resistant *Escherichia Coli*, *Klebsiella Pneumoniae*, and *Acinetobacter Baumannii*. Pharmaceutics.

[104] Allemailem K.S., Almatroudi A., Alrumaihi F., Aljaghwani A., Alnuqaydan A.M., Khalilullah H., Younus H., El-Kady A.M., Aldakheel F.M., Khan A.A. (2021). Antimicrobial, Immunomodulatory and Anti-Inflammatory Potential of Liposomal Thymoquinone: Implications in the Treatment of Bacterial Pneumonia in Immunocompromised Mice. Biomedicines.

[105] Pourhajibagher M., Partoazar A., Alaeddini M., Etemad-Moghadam S., Bahador A. (2020). Photodisinfection Effects of Silver Sulfadiazine Nanoliposomes Doped-Curcumin on *Acinetobacter Baumannii*: A Mouse Model. Nanomedicine.

[106] Ekonomou S.I., Akshay Thanekar P., Lamprou D.A., Weaver E., Doran O., Stratakos A.C. (2022). Development of Geraniol-Loaded Liposomal Nanoformulations against *Salmonella* Colonization in the Pig Gut. J. Agric. Food Chem..

[107] Engel J.B., Heckler C., Tondo E.C., Daroit D.J., da Silva Malheiros P. (2017). Antimicrobial Activity of Free and Liposome-Encapsulated Thymol and Carvacrol against *Salmonella* and *Staphylococcus Aureus* Adhered to Stainless Steel. Int. J. Food Microbiol..

[108] Ye T., Sun S., Sugianto T.D., Tang P., Parumasivam T., Chang Y.K., Astudillo A., Wang S., Chan H.-K. (2018). Novel Combination Proliposomes Containing Tobramycin and Clarithromycin Effective against *Pseudomonas Aeruginosa* Biofilms. Int. J. Pharm..

[109] Zerillo L., Polvere I., Varricchio R., Madera J.R., D’Andrea S., Voccola S., Franchini I., Stilo R., Vito P., Zotti T. (2022). Antibiofilm and Repair Activity of Ozonated Oil in Liposome. Microb. Biotechnol..

[110] Cui H., Zhang C., Li C., Lin L. (2020). Inhibition of Escherichia Coli O157:H7 Biofilm on Vegetable Surface by Solid Liposomes of Clove Oil. LWT.

[111] Montefusco-Pereira C.V., Formicola B., Goes A., Re F., Marrano C.A., Mantegazza F., Carvalho-Wodarz C., Fuhrmann G., Caneva E., Nicotra F. (2020). Coupling Quaternary Ammonium Surfactants to the Surface of Liposomes Improves Both Antibacterial Efficacy and Host Cell Biocompatibility. Eur. J. Pharm. Biopharm..

[112] Ding T., Li T., Li J. (2018). Impact of Curcumin Liposomes with Anti-Quorum Sensing Properties against Foodborne Pathogens Aeromonas Hydrophila and *Serratia Grimesii*. Microb. Pathog..

[113] Ding T., Li T., Wang Z., Li J. (2017). Curcumin Liposomes Interfere with Quorum Sensing System of Aeromonas Sobria and in Silico Analysis. Sci. Rep..

[114] Zhou Z., Hu F., Hu S., Kong M., Feng C., Liu Y., Cheng X., Ji Q., Chen X. (2018). PH-Activated Nanoparticles with Targeting for the Treatment of Oral Plaque Biofilm. J. Mater. Chem. B.

[115] Hu F., Zhou Z., Xu Q., Fan C., Wang L., Ren H., Xu S., Ji Q., Chen X. (2019). A Novel PH-Responsive Quaternary Ammonium Chitosan-Liposome Nanoparticles for Periodontal Treatment. Int. J. Biol. Macromol..

[116] Jubeh B., Breijyeh Z., Karaman R. (2020). Resistance of Gram-Positive Bacteria to Current Antibacterial Agents and Overcoming Approaches. Molecules.

[117] Khatoon Z., McTiernan C.D., Suuronen E.J., Mah T.-F., Alarcon E.I. (2018). Bacterial Biofilm Formation on Implantable Devices and Approaches to Its Treatment and Prevention. Heliyon.

[118] Oliva A., Stefani S., Venditti M., Di Domenico E.G. (2021). Biofilm-Related Infections in Gram-Positive Bacteria and the Potential Role of the Long-Acting Agent Dalbavancin. Front. Microbiol..

[119] Vazquez-Guillamet C., Kollef M.H. (2014). Treatment of Gram-Positive Infections in Critically Ill Patients. BMC Infect. Dis..

[120] Lebeaux D., Ghigo J.-M., Beloin C. (2014). Biofilm-Related Infections: Bridging the Gap between Clinical Management and Fundamental Aspects of Recalcitrance toward Antibiotics. Microbiol Mol. Biol. Rev..

[121] Römling U., Balsalobre C. (2012). Biofilm Infections, Their Resilience to Therapy and Innovative Treatment Strategies. J. Intern. Med..

[122] Cha J.-O., Yoo J.I., Yoo J.S., Chung H.-S., Park S.-H., Kim H.S., Lee Y.S., Chung G.T. (2013). Investigation of Biofilm Formation and Its Association with the Molecular and Clinical Characteristics of Methicillin-Resistant *Staphylococcus Aureus*. Osong Public Health Res. Perspect..

[123] Leshem T., Schnall B.-S., Azrad M., Baum M., Rokney A., Peretz A. (2022). Incidence of Biofilm Formation among MRSA and MSSA Clinical Isolates from Hospitalized Patients in Israel. J. Appl. Microbiol..

[124] Neyra R.C., Frisancho J.A., Rinsky J.L., Resnick C., Carroll K.C., Rule A.M., Ross T., You Y., Price L.B., Silbergeld E.K. (2014). Multidrug-Resistant and Methicillin-Resistant *Staphylococcus Aureus* (MRSA) in Hog Slaughter and Processing Plant Workers and Their Community in North Carolina (USA). Environ. Health Perspect..

[125] Piechota M., Kot B., Frankowska-Maciejewska A., Grużewska A., Woźniak-Kosek A. (2018). Biofilm Formation by Methicillin-Resistant and Methicillin-Sensitive *Staphylococcus Aureus* Strains from Hospitalized Patients in Poland. BioMed Res. Int..

[126] Wang Z., Liu X., Peng Y., Su M., Zhu S., Pan J., Shen B., Duan Y., Huang Y. (2020). Platensimycin-Encapsulated Liposomes or Micelles as Biosafe Nanoantibiotics Exhibited Strong Antibacterial Activities against Methicillin-Resistant *Staphylococcus Aureus* Infection in Mice. Mol. Pharm..

[127] Allegrone G., Ceresa C., Rinaldi M., Fracchia L. (2021). Diverse Effects of Natural and Synthetic Surfactants on the Inhibition of Staphylococcus Aureus Biofilm. Pharmaceutics.

[128] Bhatia E., Sharma S., Jadhav K., Banerjee R. (2021). Combinatorial Liposomes of Berberine and Curcumin Inhibit Biofilm Formation and Intracellular Methicillin Resistant *Staphylococcus Aureus* Infections and Associated Inflammation. J. Mater. Chem. B.

[129] Tigabu B., Getachew A. (2022). Treatment of Antibiotic-Resistant Bacteria by Nanoparticles: Current Approaches and Prospects. Ann Adv Chem.

[130] Inamuddin, Ahamed M.I., Prasad R. (2021). Microbial Biosurfactants: Preparation, Properties and Applications.

[131] Giordani B., Costantini P.E., Fedi S., Cappelletti M., Abruzzo A., Parolin C., Foschi C., Frisco G., Calonghi N., Cerchiara T. (2019). Liposomes Containing Biosurfactants Isolated from *Lactobacillus Gasseri Exert* Antibiofilm Activity against Methicillin Resistant *Staphylococcus Aureus* Strains. Eur. J. Pharm. Biopharm..

[132] Omolo C.A., Hassan D., Devnarain N., Jaglal Y., Mocktar C., Kalhapure R.S., Jadhav M., Govender T. (2021). Formulation of PH Responsive Multilamellar Vesicles for Targeted Delivery of Hydrophilic Antibiotics. Colloids Surf. B Biointerfaces.

[133] Alavi S.E., Koohi M.E.M., Raza A., Adelnia H., Ebrahimi Shahmabadi H. (2022). PEG-Grafted Liposomes for Enhanced Antibacterial and Antibiotic Activities: An in Vivo Study. NanoImpact.

[134] Ferreira M., Pinto S.N., Aires-da-Silva F., Bettencourt A., Aguiar S.I., Gaspar M.M. (2021). Liposomes as a Nanoplatform to Improve the Delivery of Antibiotics into *Staphylococcus Aureus* Biofilms. Pharmaceutics.

[135] Sulahian T.H., Imrich A., DeLoid G., Winkler A.R., Kobzik L. (2008). Signaling Pathways Required for Macrophage Scavenger Receptor-Mediated Phagocytosis: Analysis by Scanning Cytometry. Respir. Res..

[136] Li D., Chen S., Dou H., Wu W., Liu Q., Zhang L., Shen Y., Shu G., Yuan Z., Lin J. (2019). Preparation of Cefquinome Sulfate Cationic Proliposome and Evaluation of Its Efficacy on *Staphylococcus Aureus* Biofilm. Colloids Surf. B Biointerfaces.

[137] Gupta P.V., Nirwane A.M., Belubbi T., Nagarsenker M.S. (2017). Pulmonary Delivery of Synergistic Combination of Fluoroquinolone Antibiotic Complemented with Proteolytic Enzyme: A Novel Antimicrobial and Antibiofilm Strategy. Nanomed. Nanotechnol. Biol. Med..

[138] Jiang L., Li Y., Wang L., Guo J., Liu W., Meng G., Zhang L., Li M., Cong L., Sun M. (2021). Recent Insights into the Prognostic and Therapeutic Applications of Lysozymes. Front. Pharmacol..

[139] Gupta P.V., Nirwane A.M., Nagarsenker M.S. (2018). Inhalable Levofloxacin Liposomes Complemented with Lysozyme for Treatment of Pulmonary Infection in Rats: Effective Antimicrobial and Antibiofilm Strategy. AAPS PharmSciTech.

[140] Alvi S.B., Rajalakshmi P.S., Jogdand A., Sanjay A.Y., John R., Rengan A.K. (2021). Iontophoresis Mediated Localized Delivery of Liposomal Gold Nanoparticles for Photothermal and Photodynamic Therapy of Acne. Biomater. Sci..

[141] Portilla S., Fernández L., Gutiérrez D., Rodríguez A., García P. (2020). Encapsulation of the Antistaphylococcal Endolysin LysRODI in PH-Sensitive Liposomes. Antibiotics.

[142] Rout B., Liu C.-H., Wu W.-C. (2017). Photosensitizer in Lipid Nanoparticle: A Nano-Scaled Approach to Antibacterial Function. Sci. Rep..

[143] Raj V., Kim Y., Kim Y.-G., Lee J.-H., Lee J. (2022). Chitosan-Gum Arabic Embedded Alizarin Nanocarriers Inhibit Biofilm Formation of Multispecies Microorganisms. Carbohydr. Polym..

[144] Bazrgari F., Khameneh B., Bazzaz B.S.F., Mahmoudi A., Bizhan M.-N. (2020). Effect of the Nanoliposomal Formulations of Rifampin and N-Acetyl Cysteine on Staphylococcus Epidermidis Biofilm. Nanomed. J..

[145] Eroğlu İ., Aslan M., Yaman Ü., Gultekinoglu M., Çalamak S., Kart D., Ulubayram K. (2020). Liposome-Based Combination Therapy for Acne Treatment. J. Liposome Res..

[146] Natsaridis E., Gkartziou F., Mourtas S., Stuart M.C.A., Kolonitsiou F., Klepetsanis P., Spiliopoulou I., Antimisiaris S.G. (2022). Moxifloxacin Liposomes: Effect of Liposome Preparation Method on Physicochemical Properties and Antimicrobial Activity against Staphylococcus Epidermidis. Pharmaceutics.

[147] Gerayelou G., Khameneh B., Malaekeh-Nikouei B., Mahmoudi A., Fazly Bazzaz B.S. (2020). Dual Antibiotic and Diffusible Signal Factor Combination Nanoliposomes for Combating *Staphylococcus Epidermidis* Biofilm. Adv. Pharm. Bull..

[148] Francolini I., Giansanti L., Piozzi A., Altieri B., Mauceri A., Mancini G. (2019). Glucosylated Liposomes as Drug Delivery Systems of Usnic Acid to Address Bacterial Infections. Colloids Surf. B Biointerfaces.

[149] Vögeling H., Plenagl N., Seitz B.S., Duse L., Pinnapireddy S.R., Dayyoub E., Jedelska J., Brüßler J., Bakowsky U. (2019). Synergistic Effects of Ultrasound and Photodynamic Therapy Leading to Biofilm Eradication on Polyurethane Catheter Surfaces Modified with Hypericin Nanoformulations. Mater. Sci. Eng. C.

[150] Plenagl N., Seitz B.S., Duse L., Pinnapireddy S.R., Jedelska J., Brüßler J., Bakowsky U. (2019). Hypericin Inclusion Complexes Encapsulated in Liposomes for Antimicrobial Photodynamic Therapy. Int. J. Pharm..

[151] Weiser J.N., Ferreira D.M., Paton J.C. (2018). *Streptococcus Pneumoniae*: Transmission, Colonization and Invasion. Nat. Rev. Microbiol..

[152] Chao Y., Marks L.R., Pettigrew M.M., Hakansson A.P. (2015). *Streptococcus Pneumoniae* Biofilm Formation and Dispersion during Colonization and Disease. Front. Cell. Infect. Microbiol..

[153] Silva M.D., Paris J.L., Gama F.M., Silva B.F.B., Sillankorva S. (2021). Sustained Release of a *Streptococcus Pneumoniae* Endolysin from Liposomes for Potential Otitis Media Treatment. ACS Infect. Dis..

[154] Love M., Bhandari D., Dobson R., Billington C. (2018). Potential for Bacteriophage Endolysins to Supplement or Replace Antibiotics in Food Production and Clinical Care. Antibiotics.

[155] Ingendoh-Tsakmakidis A., Eberhard J., Falk C.S., Stiesch M., Winkel A. (2020). In Vitro Effects of *Streptococcus Oralis* Biofilm on Peri-Implant Soft Tissue Cells. Cells.

[156] Matsumoto-Nakano M. (2018). Role of Streptococcus Mutans Surface Proteins for Biofilm Formation. Jpn. Dent. Sci. Rev..

[157] Fang J.-Y., Chou W.-L., Lin C.-F., Sung C.T., Alalaiwe A., Yang S.-C. (2021). Facile Biofilm Penetration of Cationic Liposomes Loaded with DNase I/Proteinase K to Eradicate *Cutibacterium Acnes* for Treating Cutaneous and Catheter Infections. Int. J. Nanomed..

[158] Drost M., Diamanti E., Fuhrmann K., Goes A., Shams A., Haupenthal J., Koch M., Hirsch A.K.H., Fuhrmann G. (2021). Bacteriomimetic Liposomes Improve Antibiotic Activity of a Novel Energy-Coupling Factor Transporter Inhibitor. Pharmaceutics.

[159] Blanchard J.D., Elias V., Cipolla D., Gonda I., Bermudez L.E. (2018). Effective Treatment of *Mycobacterium Avium* Subsp. *Hominissuis* and *Mycobacterium Abscessus* Species Infections in Macrophages, Biofilm, and Mice by Using Liposomal Ciprofloxacin. Antimicrob. Agents Chemother..

[160] Zhang J., Leifer F., Rose S., Chun D.Y., Thaisz J., Herr T., Nashed M., Joseph J., Perkins W.R., DiPetrillo K. (2018). Amikacin Liposome Inhalation Suspension (ALIS) Penetrates Non-Tuberculous Mycobacterial Biofilms and Enhances Amikacin Uptake into Macrophages. Front. Microbiol..

[161] Pu C., Tang W. (2017). The Antibacterial and Antibiofilm Efficacies of a Liposomal Peptide Originating from Rice Bran Protein against *Listeria Monocytogenes*. Food Funct..

[162] Ruiz A., Herráez M., Costa-Gutierrez S.B., Molina-Henares M.A., Martínez M.J., Espinosa-Urgel M., Barriuso J. (2021). The Architecture of a Mixed Fungal–Bacterial Biofilm Is Modulated by Quorum-sensing Signals. Environ. Microbiol..

[163] Allkja J., Azevedo A.S., Azevedo N.F., Almeida C. (2021). Characterization of Social Interactions and Spatial Arrangement of Individual Bacteria in MultiStrain or Multispecies Biofilm Systems Using Nucleic Acid Mimics-Fluorescence in situ Hybridization. Fluorescence In Situ Hybridization (FISH) for Microbial Cells.

[164] Chitlapilly Dass S., Bosilevac J.M., Weinroth M., Elowsky C.G., Zhou Y., Anandappa A., Wang R. (2020). Impact of Mixed Biofilm Formation with Environmental Microorganisms on *E. Coli* O157:H7 Survival against Sanitization. npj Sci. Food..

[165] Alrbyawi H., Poudel I., Annaji M., Arnold R.D., Tiwari A.K., Babu R.J. (2022). Recent Advancements of Stimuli-Responsive Targeted Liposomal Formulations for Cancer Drug Delivery. Pharm. Nanotechnol..

[166] Estelrich J., Busquets M. (2018). Iron Oxide Nanoparticles in Photothermal Therapy. Molecules.

[167] Shipunova V.O., Belova M.M., Kotelnikova P.A., Shilova O.N., Mirkasymov A.B., Danilova N.V., Komedchikova E.N., Popovtzer R., Deyev S.M., Nikitin M.P. (2022). Photothermal Therapy with HER2-Targeted Silver Nanoparticles Leading to Cancer Remission. Pharmaceutics.

[168] Sun P., Ye L., Tan X., Peng J., Zhao L., Zhou Y. (2022). Silver Nanoparticle-Assisted Photodynamic Therapy for Biofilm Eradication. ACS Appl. Nano Mater..

